# The Genetics of Symbiotic Nitrogen Fixation: Comparative Genomics of 14 Rhizobia Strains by Resolution of Protein Clusters 

**DOI:** 10.3390/genes3010138

**Published:** 2012-02-16

**Authors:** Michael Black, Paula Moolhuijzen, Brett Chapman, Roberto Barrero, John Howieson, Mariangela Hungria, Matthew Bellgard

**Affiliations:** 1 Centre for Comparative Genomics, Murdoch University, South Street, Murdoch, Perth, WA 6150, Australia; E-Mails: pmoolhuijzen@ccg.murdoch.edu.au (P.M.); bchapman@ccg.murdoch.edu.au (B.C.); rbarrero@ccg.murdoch.edu.au (R.B.);; 2 Centre for Rhizobium Studies, Murdoch University, South Street, Murdoch, Perth, WA 6150, Australia; E-Mail: j.howieson@murdoch.edu.au; 3 Embrapa Soja, Cx. Postal 231, Londrina, Parana, 86001-970, Brazil; E-Mail: hungria@cnpso.embrapa.br

**Keywords:** nitrogen fixation, symbiosis, *Rhizobiales*, genome, comparative genomics, nodulation genes, secretion systems

## Abstract

The symbiotic relationship between legumes and nitrogen fixing bacteria is critical for agriculture, as it may have profound impacts on lowering costs for farmers, on land sustainability, on soil quality, and on mitigation of greenhouse gas emissions. However, despite the importance of the symbioses to the global nitrogen cycling balance, very few rhizobial genomes have been sequenced so far, although there are some ongoing efforts in sequencing elite strains. In this study, the genomes of fourteen selected strains of the order *Rhizobiales*, all previously fully sequenced and annotated, were compared to assess differences between the strains and to investigate the feasibility of defining a core ‘symbiome’—the essential genes required by all rhizobia for nodulation and nitrogen fixation. Comparison of these whole genomes has revealed valuable information, such as several events of lateral gene transfer, particularly in the symbiotic plasmids and genomic islands that have contributed to a better understanding of the evolution of contrasting symbioses. Unique genes were also identified, as well as omissions of symbiotic genes that were expected to be found. Protein comparisons have also allowed the identification of a variety of similarities and differences in several groups of genes, including those involved in nodulation, nitrogen fixation, production of exopolysaccharides, Type I to Type VI secretion systems, among others, and identifying some key genes that could be related to host specificity and/or a better saprophytic ability. However, while several significant differences in the type and number of proteins were observed, the evidence presented suggests no simple core symbiome exists. A more abstract systems biology concept of nitrogen fixing symbiosis may be required. The results have also highlighted that comparative genomics represents a valuable tool for capturing specificities and generalities of each genome.

## 1. Introduction

The symbiotic relationship between legumes and nitrogen fixing bacteria is critical for agriculture, as it may have profound impacts on lowering costs for farmers, on land sustainability, on soil quality, and on mitigation of greenhouse gas emissions. The major importance of the symbioses is usually attributed to the decrease in the use of costly nitrogen based fertilizers, but the rehabilitation of infertile, environmentally stressed soils should also be highlighted. With an increasing global demand for food production, combined with the need to reduce carbon emissions, the reliance on biological nitrogen fixation as an alternative to nitrogen fertilizers is forecast to increase [1].

Symbiotic nitrogen fixing bacteria are represented by a phylogenetically disparate class of alpha- and beta-proteobacteria—usually collectively termed rhizobia—that have achieved the function of fixing atmospheric nitrogen (N_2_) in symbiosis with legumes. The majority of the symbiotic speciesare represented in the alpha-proteobacteria order *Rhizobiales*, which, amongst many others, contain the agriculturally important nitrogen fixing genera of *Rhizobium*, *Bradyrhizobium*, *Mesorhizobium*, *Sinorhizobium* (=*Ensifer*) and *Azorhizobium*. One impediment to the broader use of rhizobia in agriculture is the production of compatible inoculants. There are substantial host, strain and environmental specificities that limit the use of potentially important legume fodder and crops as alternatives to nitrogen fertilizers [2,3]. 

Despite the importance of nitrogen fixation to the global nitrogen balance, very few rhizobial strains have been completely sequenced, representing less than 1% of the complete bacterial genomes available today. The scenario may slightly improve in the following years, as there are large-scale genome sequencing projects in progress. Furthermore, very few genomic studies comparing nitrogen fixing bacteria have been performed [4,5], but the results obtained have indicated that comparative genomics represents a promising tool to reveal bacterial specificities. Furthermore, advances in bioinformatics tools will reveal increasing details in the comparison of genomes.

In this present study, fourteen rhizobial genomes were selected based on maximizing geographical, environmental and host range, spanning most Vavilov centers of origin [6]. This comparison was undertaken to attempt to establish a reference ‘symbiome’, meaning a set of genes critical to successful symbiosis and subsequent nitrogen fixation, as well as to obtain a better understanding of the strategies adopted by disparate strains to maintain their symbiotic apparatus. Different tools were evaluated in the comparison, and the results may also contribute to a further exploration in future large scale comparative genomic studies.

## 2. Results and Discussion

### 2.1. Key Characteristics of the Fourteen Strains of the Order *Rhizobiales*

The aim of this study was to investigate the possibility of defining a common ‘symbiome’ amongst selected nitrogen fixing strains of the order *Rhizobiales*. The fourteen genomes selected represent a broad range, from classic legume symbionts to *Mesorhizobium* sp. BNC1, a strain that has seemingly lost the ability to fix nitrogen and form symbiotic relationships. In addition, the selected organisms represent both root nodulating (e.g., *Bradyrhizobium japonicum* USDA 110, symbiont of soybean—*Glycine max*, *Sinorhizobium meliloti* 1021, symbiont of alfalfa—*Medicago sativa*)) and stem nodulating (e.g., *Azorhizobium caulinodans* strain ORS571, symbiont of sesbania—*Sesbania rostrata*) microsymbionts. All available nucleotide and protein FASTA files, as well as GENBANK files available were utilized (Table 1). The fourteen genomes sequenced and their hosts are listed in Table 2, and the strains will be referred to by their KEGG organism code.

#### 2.1.1. Azorhizobium

*A. caulinodans* (*azc*) is a nitrogen fixing member of the *Xanthobacteraceae* family. It is primarily a stem nodulator of the African legume sesbania. It is thought to have originally been a non-nitrogen fixer that developed the ability to fix N_2_ entirely through lateral gene transfer from another unknown species [7]. Unlike the other thirteen strains from this study, *azc* can reduce di-nitrogen both in symbiosis and in the free living stage, and as a result can be grown on nitrogen free medium, a key defining property. It has a relatively small genome—5.37 MB—in comparison to other rhizobia and it is the most taxonomically distant species in this study [7,8].

#### 2.1.2. Bradyrhizobium

Three *Bradyrhizobium* genomes have been selected, *B. japonicum* USDA 110 (*bja*), *Bradyrhizobium* sp. BTAi1 (*bbt*), and *Bradyrhizobium* ORS278 (*bra*). *Bradyrhizobium* is primarily distinguished from the genera *Mesorhizobium, Rhizobium* and *Sinorhizobium* by the slower growth rate (doubling time) of at least 8 h.

The soybean root nodulator *bja* has the largest genome of this study, 9.1 MB, in a unique chromosome [9]. The main feature of the more recently obtained genomes of *Bradyrhizobium* strains BTAi1 (*bbt*) and ORS278 (*bra*) is that they are photosynthetic stem nodulators of tropical *Aeschynomene* species, and for the first time described as lacking common nodulation genes *nodA*, *nodB* and *nodC.* These genes are traditionally involved in the construction of the Nod factor backbone [10,11]. Both strains also have large genomes, similar to *bja* (Table 2).

**Table 1:** REFSEQ identifiers for the fourteen *Rhizobiales* genomes.

*Rhizobium* species	Chr	Plasmid1	Plasmid2	Plasmid3	Plasmid4	Plasmid5	Plasmid6
*Azorhizobium caulinodans* ORS 571	NC_009937	-	-	-	-	-	-
*Bradyrhizobium japonicum* USDA 110	NC_004463	-	-	-	-	-	-
*Bradyrhizobium* sp. BTAi1	NC_009485	NC_009475					
*Bradyrhizobium* sp. ORS 278	NC_009445	-	-	-	-	-	-
*Mesorhizobium loti* MAFF303099	NC_002678	NC_002679	NC_002682				
*Mesorhizobium* sp. BNC1 (*Chelativorans* sp. BNC1)	NC_008254	NC_008242	NC_008243	NC_008244			
*Rhizobium etli* CFN 42	NC_007761	NC_007762	NC_007763	NC_007764	NC_004041	NC_007765	NC_007766
*Rhizobium etli* CIAT 652	NC_010994	NC_010998	NC_010996	NC_010997			
*Rhizobium leguminosarum bv. trifolii* WSM1325	NC_012850	NC_012848	NC_012858	NC_012853	NC_012852	NC_012854	
*Rhizobium leguminosarum bv. trifolii* WSM2304	NC_011369	NC_011368	NC_011366	NC_011370	NC_011371		
*Rhizobium leguminosarum bv. viciae* 3841	NC_008380	NC_008382	NC_008383	NC_008379	NC_008381	NC_008384	NC_008378
*Sinorhizobium fredii* sp. NGR 234	NC_012587	NC_000914	NC_012586				
*Sinorhizobium medicae* WSM419	NC_009636	NC_009620	NC_009621	NC_009622			
*Sinorhizobium meliloti* 1021	NC_003047	NC_003037	NC_003078				

**Table 2:** Summary statistics of the fourteen genomes of *Rhizobiales*.

*Rhizobiales* species	Species Code	No. of Plasmids	Total Genome Length (nucleotides)	Protein Coding Genes	tRNA genes	Pseudo genes	GC Content (%)	Proportion of Genome that is Gene Coding (%)	Host (Scientific/common name)
*Azorhizobium caulinodans* ORS 571	*azc*	0	5,369,772	4717	63	-	67	89	*Sesbania rostrata (sesbania)*
*Bradyrhizobium japonicum* USDA 110	*bja*	0	9,105,828	8317	56	-	64	86	*Glycine max (soybean)*
*Bradyrhizobium* sp. BTAi1	*bbt*	1	8,493,513	7621	70	90	64	85	*Aeschynomene indica (Indian joint-vetch)*
*Bradyrhizobium* sp. ORS 278	*bra*	0	7,456,587	6717	66	35	65	85	*Aeschynomene sensitiva (sensitive joint-vetch)*
*Mesorhizobium loti* MAFF303099	*mlo*	2	7,596,297	7272	57	-	62	86	*Lotus* sp., *including Lotus japonicas (trifoils, vetches)*
*Mesorhizobium* sp. BNC1	*mes*	3	4,935,185	4543	68	40	61	89	*non-symbiotic*
*Rhizobium etli* CFN 42	*ret*	6	6,530,228	5963	59	32	61	86	*Phaseolus vulgaris (common bean)*
*Rhizobium etli* CIAT 652	*rec*	3	6,448,048	6056	60	15	61	86	*Phaseolus vulgaris (common bean)*
*Rhizobium leguminosarum bv. trifolii* WSM1325	*rlg*	5	7,418,122	7001	63	75	61	86	*Trifolli pratense and other Mediterraneum Trifollium* (clovers),
*Rhizobium leguminosarum bv. trifolii* WSM2304	*rlt*	4	6,872,702	6415	65	45	61	86	*Trifolium polymorphum* from Uruguay (clover)
*Rhizobium leguminosarum bv. viciae* 3841	*rle*	6	7,751,309	7143	61	37	61	86	Tribe Viciae –*Vicia, Pisum, Lathyrus, Lens* (vetchs, peas, lathyrus, lentils)
*Sinorhizobium fredii* NGR 234	*rhi*	2	6,891,900	6363	70	-	63	87	112 legume species and the non-legume *Parasponia* (family Ulmaceae)
*Sinorhizobium medicae* WSM419	*smd*	3	6,817,576	6213	63	43	61	87	*Medicago* spp.
*Sinorhizobium meliloti* 1021	*sme*	2	6,691,694	6218	66	4	62	86	*Medicago, Melilotus, Trigonella (alfalfa)*

#### 2.1.3. Mesorhizobium

As the suffix *Meso* suggests, species of this genus show growth rates intermediate to *Bradyrhizobium* (>8 h) and *Rhizobium*/*Sinorhizobium* (<6 h). *Mesorhizobium loti* MAFF303099 (*mlo*) is a root nodulator of *Lotus japonicum*, with one chromosome and two plasmids; as in other *Mesorhizobium* and *Bradyrhizobium*, *mlo* has a symbiotic island within the chromosome that contains all key genes for nodulation and nitrogen fixation [12].

*Mesorhizobium* sp. BNC1 (*mes*), formerly known as *Agrobacterium* sp. BNC1, and alternately named *Chelativorans* sp. BNC1, is the functional outlier of the fourteen species, being asymbiotic. It was isolated from a mixed-culture enriched from sewage using the chelating agent EDTA as the sole carbon and nitrogen source [13]. For this study, *mes* is utilized as an outlier for the basis of “symbiome” definition and investigation.

#### 2.1.4. Rhizobium

The genus *Rhizobium*, originally defining all bacterium with the ability to nodulate legumes [14], has undergone multiple redefinitions and now encompasses a variety of fast growing nitrogen fixers including some former *Agrobacterium* and *Sinorhizobium* (*Ensifer)* species and the genus *Allorhizobium*. In this study, five *Rhizobium* genomes are investigated: *R. etli* CFN 42 (*ret*), *R. etli* CIAT 652 (*rec*), *R. leguminosarum* bv. *trifolii* WSM1325 (*rlg*), *R. leguminosarum* bv. *trifolii* WSM2304 (*rlt*) and *R. leguminosarum* bv. *viciae* 3841 (*rle*). These five *Rhizobium* strains have different origins and hosts. The *R.* *etli* strains nodulate common bean (*Phaselous vulgaris* and were originally isolated in Central America) [15,16,17,18]. In contrast, the *R.* *leguminosarum* strains nodulate temperate legumes: bv. trifolii *rlg* and *rlt* are symbionts of clovers (*Trifolium*), although individually matched to annual (*rlg*) and perennial (*rlt*) species for N fixation [19,20], Bv. viciae *rle* has a broader host range, nodulating legumes within the tribe *Viciae*, including *Pisum, Vicia, Lathyrus* and *Lens* [21].

#### 2.1.5. Sinorhizobium

The genus *Sinorhizobium* is subject of some controversy. Members of this genus were originally classified as *Rhizobium* (e.g., *Rhizobium meliloti*) and the new genus was proposed in 1988 [22]. More recently, the genus has been reclassified as *Ensifer* [23,24], but in general the new nomenclature was not broadly accepted by rhizobiologists. *Sinorhizobium* and *Rhizobium* have similar morpho-physiological properties, and only the 16S rRNA taxonomy resolves the genus as a distinct clade [25].

Three *Sinorhizobium* were chosen for this study, *S. meliloti* 1021 (*sme*), *S.* *medicae* WSM419 (*smd*) and *Sinorhizobium* sp. strain NGR 234 (*rhi*), also denominated as *S. fredii* NGR 234 *and Rhizobium* sp. NGR 234 [26,27,28]. Both *sme* and *smd* nodulate temperate legumes of the genus *Medicago*. It has been established that *smd* has greater acid tolerance, and is a more effective nodulator of *Medicago truncatula* than *sme* [29,30]. It is also hypothesized that *sme* and *smd* evolved in association with hosts adapted to different edaphic conditions [31]. The tropical strain NGR 234 (*rhi)* is probably the most intriguing rhizobia isolated so far. Originally isolated in Papua New Guinea and labeled *Rhizobium* sp. NGR 234, it has since been found to be highly promiscuous, capable of nodulating at least 112 different legume genera, from most Vavilov centers of origin, in addition to the non-legume *Parasponia* [32,33,34]. 

### 2.2. Phylogeny and Taxonomy of Rhizobiales

#### 2.2.1. 16S rRNA Taxonomy

The phylogeny and taxonomy of nitrogen fixing *Rhizobiales* is in a state of flux. Confusion over the status of *Sinorhizobium* [22,23,35] and the split phylogeny of *Agrobacterium* within the genus *Rhizobium* [24,36] are just two cases in point. Therefore in this genomic comparison several methods of taxonomic and genomic comparison were utilized to highlight different aspects of the phylogeny of the selected strains. 

**Figure 1:**
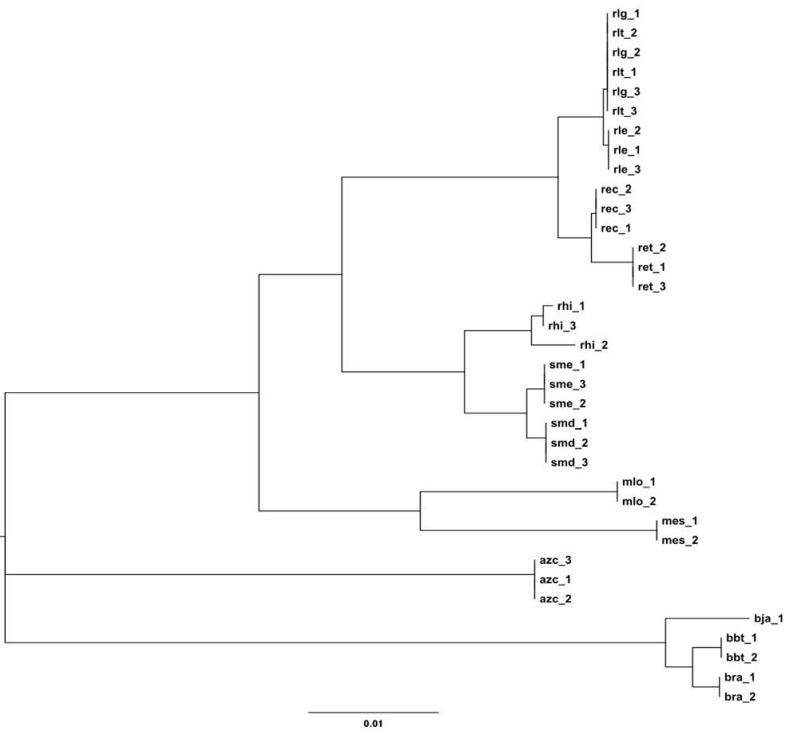
16S rRNA gene tree built with the multiple 16S rRNA genes from each of the fourteen genomes compared. Taxonomic analysis performed with MEGA5 [68], by constructing a bootstrapped neighbor-joining (NJ) gene tree with Jukes-Cantor substitution.

The ‘classical’ relationship between *Bradyrhizobium*, *Rhizobium*, *Sinorhizobium* and *Mesorhizobium* is clearly shown in the 16S rRNA phylogeny (Figure 1). The four major groups are delineated from each other forming four major clusters, with *Azorhizobium* as a separate branch. All strains were clustered within the expected genera and as expected the *Trifolium* nodulators *rlg* and *rlt* were indistinguishable from each other using exclusively the 16S rRNA. 

#### 2.2.2. Dotplot Analysis

**Figure 2:**
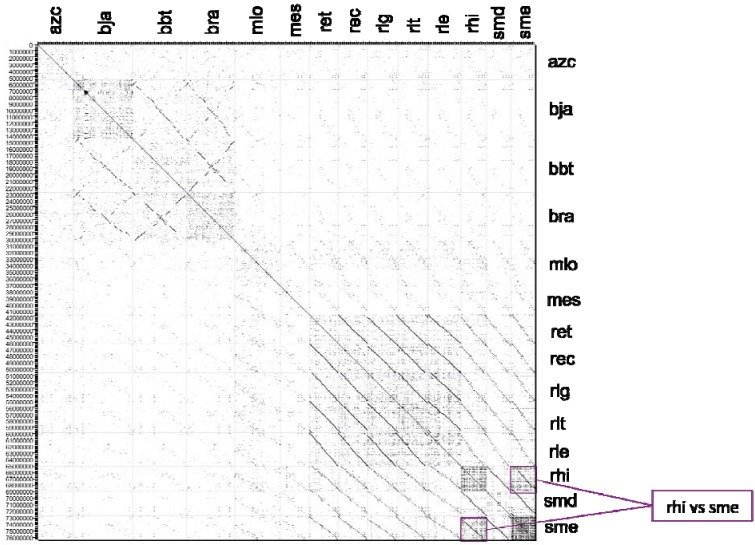
FRECKLE DNA dotplot of the fourteen *Rhizobiales* chromosomes, constructed from nucleotide FASTA files using an in house script.

Further comparison of whole chromosomes adds valuable information to the 16S rRNA taxonomy results. A dotplot of the chromosome nucleotide sequences was compiled using an in-house application called FRECKLE (Figure 2) (http://code.google.com/p/freckle/). Two primary blocks of significant sequence similarity are those of the *Bradyrhizobium* species with another larger block consisting of the *Rhizobium* and *Sinorhizobium* strains, while *Azorhizobium* and *Mesorhizobium* are outliers. The *sme* chromosome shows a highly repetitive structure, more than any other chromosome. Interestingly, the dotplot also displayed a strong sequence similarity between *Sinorhizobium* NGR 234 and *S. meliloti* 1021, including a shared repetitive structure. 

A dotplot of plasmids (Figure S1) shows markedly less homology. Greater symmetries were detected in *Rhizobium* non-symbiotic plasmids: plasmids p42e (*R. etli* CFN 42), pRL11 (*R. leguminosarum* bv. viciae 3841), pRLG202 (*R. leguminosarum* bv. trifolii WSM2304) and pR132502 (*R. leguminosarum* bv. trifolii WSM132502) are especially homologous, suggesting higher stability. In addition, pSMED01 (*S. medicae* WSM419) and pSymB (*S. meliloti* 1021) also show symmetry. This observation has been previously explored, resulting in the proposal that these plasmids—with little or no direct involvement in the symbiosis—represent secondary chromosomes [37], or alternatively ‘chromids’ [38], as they contain essential metabolic genes, as well as tRNA and/or rRNA. 

In contrast, little homology was detected in the analysis of the symbiotic plasmids (Figure S1). Symbiotically important plasmids have similar *nod*, *nif* and, in some cases, *fix* clusters. It is hypothesized that multiple lateral transfer events, which originally enabled the symbiotic and nitrogen fixing ability of many of these bacteria, would have also caused the overall lack of homology amongst the symbiotic plasmids. Our study has pointed out that the *rhi* plasmids pNGR234a and pNGR234b display lesser homology with the other plasmids, suggesting a greater number of lateral transfer events. Such ‘elastic’ genomes could partly explain the adaptation to a wide host range of *rhi*. 

The contrast between the 16S rRNA taxonomy and the two dotplots (chromosomal and plasmids) relies on the disparate relationships between the plasmids. In fact, Young *et al.* 2006 concluded that *R. leguminosarum* bv. viciae (*rle*) had a core genome, mostly chromosomal with a high G+C content and shared with other organisms, and an accessory genome with a lower G+C content, on plasmids and chromosomal genomic islands [21]. The broader whole genome taxonomy in this study suggests that this two-component genome model may prevail in other rhizobial species. The *R.* *etli* and *R.* *leguminosarum* bv. trifolii genomes species share much of their chromosome with *R.* *leguminosarum* bv. viciae, further expanding the “core—genome” definition of Young *et al.* 2006, and, in turn, shrinking the “accessory genome”. 

Most important, the low homology observed in this study in the comparison of the genomes of both closely related strains within the same species and also between species showing reasonable similarity of the 16S rRNA, and clearly showing several events of lateral gene transfer reinforces the discussion about the validity of prokaryote species definition [39,40]. 

### 2.3. KEGG Orthology and Protein Clustering

#### 2.3.1. KEGG Pathway Analysis

The next stage of our genomic study was to compare KEGG genes and pathways in each species. The summary of KEGG orthologs is shown in Table 3, and the full table of KEGG pathways is included as Table S1.

All fourteen genomes have a large number of nitrogen, methane, sulfur, amino acid, vitamins and cofactors metabolism KEGG orthologs. This diverse suite of metabolism pathways is indicative of the ability to live in the complex rhizosphere environments, as well as to adapt to the nodule environment. However, the fact that the non-symbiotic genome of *mes* shows the same wide range of KEGG metabolism pathway orthologs as the symbiotic *mlo*, indicates the role of these genes in determining saprophytic capacity in a broad range of soil conditions. Noteworthy was the wide range of nitrogen metabolism protein orthologs detected (Table S2), showing a variety of nitrogen metabolism capabilities. The largest number of orthologs in *Bradyrhizobium* and *Sinorhizobium* indicate the capacity to metabolize nitriles, nitrates, formamide, nitroalkanes, as well as related amino acids glutamine and asparagine. 

The genomes of *Mesorhizobium* and *Rhizobium leguminosarum* lack *norC*, *norD* and *norE* orthologs, protein subunits of the nitric oxide reductase complex. Interestingly, within the same species *R. etli*, *ret* but not *rec* contains asparagine synthetase (*asnB)* and *nor* orthologs. *R. etli* CFN 42 (*ret*) is unable to use nitrate for respiration and lacks nitrate reductase activity, as well as the *nap* and *nar* genes encoding respiratory nitrate reductase; however, the strain carries proteins closely related to denitrification enzymes, *norCBQD*. The functionality of *nor* genes in CFN 42 has been recently demonstrated, allowing the reduction of nitrite and nitric oxide [41]. It is worth mentioning that differences between strains within the same species made visible only by comparative genomics can help to explain metabolomic advantages, e.g., in this case of *ret* over *rec*.

#### 2.3.2. Protein Cluster Analysis

A comprehensive method of comparative genome analysis is to cluster protein families with the BlastlineMCL algorithm [42], representing a Markov clustering of all orthologous protein groups across species. This could be considered a more complete clustering of orthologous proteins than from KEGG, as it includes all proteins annotated across all the genomes, not just the more restricted set of established KEGG orthologs. The use of this tool has been shown to be crucial in the comparison of rhizobial species, as many symbiotic and nitrogen fixing related gene pathways and families are not represented in KEGG.

Cluster information resolved from BlastlineMCL analysis is available online (see experimental section), where users can compare any combination of genome protein family homology to search for orthologous protein groups of interest. Raw sequence data is available in FASTA format and CLUSTALW aligned format (MSF). This resource represents a vast repository of comparative genomics information on the fourteen genomes, of which only a few are discussed in this paper.

A nitrogen-fixing *Rhizobiales* “pan-genome” of 1,126 clusters and 28,110 proteins was resolved amongst the fourteen genomes (Table 4). The clusters within this group include ABC transporters, some transposases, ribosomal RNA synthetases, DNA polymerases and other core proteins. The ABC transporters are the second most abundant family of non-hypothetical protein encoding genes found in all sequenced prokaryotic, eukaryotic, viral genomes as well as in metagenomic sequences. ABC transporters represent one of the largest superfamilies of active membrane transport proteins (MTPs), with a highly conserved ATPase domain that binds and hydrolyzes ATP, supplying energy for the uptake of a variety of nutrients and for the extrusion of drugs and metabolic wastes [43]. It is therefore not surprising that by far the biggest cluster found in the BlastlineMCL analysis is that of ABC type transporters, with 1,128 separate proteins. Together with the previous KEGG findings, this is indicative of ability to use a very wide range of substrates, a key property for adaption in the variety of environments, such as the numerous geographical rhizospheres represented (such as China, Papua New Guinea, Uruguay and Mexico) and the diverse hosts they nodulate.

**Table 3:** Summary of KEGG pathway and protein orthologs in the fourteen *Rhizobiales* genomes.

Relevant KEGG Pathways	Number of KEGG Protein Orthologs													
	azc	bja	bbt	bra	mlo	mes	ret	rec	rlg	rlt	rle	rhi	smd	sme
1.1 Carbohydrate Metabolism	240	295	295	296	274	245	249	279	257	256	299	262	267	273
1.2 Energy Metabolism	114	147	146	145	113	114	112	111	99	99	118	111	124	138
1.3 Lipid Metabolism	52	63	72	72	72	53	61	76	58	57	81	56	58	71
1.4 Nucleotide Metabolism	102	96	100	99	111	105	100	108	97	99	112	97	98	114
1.5 Amino Acid Metabolism	221	249	268	258	275	224	235	261	227	241	272	239	236	253
1.6 Metabolism of Other Amino Acids	50	58	62	57	60	48	54	58	54	57	58	56	54	62
1.7 Glycan Biosynthesis and Metabolism	30	29	31	33	32	25	33	35	32	34	36	31	22	32
1.8 Metabolism of Cofactors and Vitamins	117	132	143	137	131	103	122	126	114	117	130	119	117	124
1.9 Biosynthesis of Polyketides and Terpenoids	29	29	42	43	29	27	28	36	28	27	34	27	27	34
1.10 Biosynthesis of Other Secondary Metabolites	8	21	32	35	31	20	27	34	26	26	37	26	23	28
1.11 Xenobiotics Biodegradation and Metabolism	85	134	166	163	97	72	58	129	60	59	138	55	53	101
2.1 Transcription	4	4	4	4	4	4	4	4	4	4	4	4	4	4
2.2 Translation	134	131	128	129	130	123	128	128	129	129	127	124	129	132
2.3 Folding, Sorting and Degradation	40	40	38	38	36	34	36	37	38	38	37	36	38	38
2.4 Replication and Repair	69	74	71	71	70	70	71	71	73	71	73	70	71	71
3.1 Membrane Transport	119	141	121	114	172	135	159	163	138	131	162	165	148	170
3.2 Signal Transduction	57	61	55	57	50	39	50	55	48	48	53	49	46	49
4.2 Cell Motility	38	41	43	43	34	38	40	41	40	40	41	40	40	40
Total KEGG Protein Orthologs	1509	1745	1817	1794	1721	1479	1567	1752	1522	1533	1812	1567	1555	1734

**Table 4:** Summary of selected MCL BLASTline protein cluster groups.

Cluster Family	Clusters	Proteins	Proteins in Chromosomes	Proteins in Plasmids	Percent in Chromosomes	Percent in Plasmids
Pan-genome (all 14)	1126	28110	23686	4424	84.26	15.74
13 Fix+ genomes	105	1126	619	507	54.97	45.03
11 NodABC+ genomes	9	113	60	53	53.10	46.90
*azc+bbt+bja+bra*	206	1150	1141	9	99.22	0.78
*bbt+bja+bra*	857	2981	2976	5	99.83	0.17
*bbt+bra*	577	1224	1224	0	100.00	0.00
*mlo+mes*	86	192	167	25	86.98	13.02
*mes+mlo+rhi+ret+rec+rlg+rlt+rle+smd+sme*	214	2424	2081	343	85.85	14.15
*mlo+rhi+ret+rec+rlg+rlt+rle+smd+sme*	161	1661	1140	521	68.63	31.37
*rhi+ret+rec+rlg+rlt+rle+smd+sme*	155	1347	1122	225	83.30	16.70
*rhi+ret+rec+rlg+rlt+rle*	51	347	191	156	55.04	44.96
*rlg+rlt+rle*	92	286	197	89	68.88	31.12
*smd+sme*	253	555	182	373	32.79	67.21
*rhi+ret+rec*	6	21	5	16	23.81	76.19
*rhi+smd+sme*	242	767	476	291	62.06	37.94
*ret+rec*	123	262	71	191	27.10	72.90
*ret+rec+rlg+rlt+rle*	352	1866	1436	430	76.96	23.04
**Singletons**						
*azc*	956	986	986	0	100.00	0.00
*bja*	1760	1839	1839	0	100.00	0.00
*bbt*	1051	1107	1005	102	90.79	9.21
*bra*	959	987	987	0	100.00	0.00
*mlo*	1706	1809	1613	196	89.17	10.83
*mes*	857	899	761	138	84.65	15.35
*ret*	333	344	196	148	56.98	43.02
*rec*	563	563	374	189	66.43	33.57
*rlg*	576	602	260	342	43.19	56.81
*rlt*	490	492	286	206	58.13	41.87
*rle*	542	550	276	274	50.18	49.82
*rhi*	758	797	356	441	44.67	55.33
*smd*	598	639	317	322	49.61	50.39
*sme*	472	498	170	328	34.14	65.86

The number of clusters shared amongst all thirteen nitrogen fixers was of 105, containing a total of 2,038 proteins. Many of these clusters are of *nif* proteins, the components of the nitrogenase complex. Nine protein clusters were shared by the eleven strains containing the *nodABC* genes, with a total of 113 proteins. These clusters include other *nod*, *nol* and *noe* gene families, protein families crucial to nodulation.

Table 4 lists many other cluster sharing combinations. For example, 857 clusters are exclusively shared by the *Bradyrhizobium* species, confirming the differences between this genus and the others. In addition, the 242 clusters containing 767 proteins—60% in chromosomes—found only in *rhi*, *smd*, *sme* confirm *rhi* as a *Sinorhizobium* species.

The number of singleton clusters (clusters containing proteins only from one species) approximately correlated with previously resolved taxonomic divisions, as well as genome size (Table 4). The largest number of singletons was found in the biggest genome, *bja* (1,760 clusters containing 1,839 proteins). The number of singletons amongst the various *Rhizobium* and *Sinorhizobium* was much smaller, ranging from 758 clusters containing 797 proteins in *rhi* to 333 clusters containing 344 proteins in *rec.* The most common protein family found amongst these singletons is composed by hypothetical proteins.

### 2.4. The “Symbiome”- Nodulation, Secretion, Exopolysaccharide Production, Oxygen Transport and Nitrogen Fixation

A precise definition of what is included in a theoretical ‘symbiome’ is still elusive. For the purposes of the current study, the core ‘symbiome’ is defined as the protein families, found in the BlastlineMCL clusters, involved in symbiosis and in nitrogen fixation. For this study this is defined as proteins for nodulation, secretion, exopolyssacharide production, oxygen transport and nitrogen fixation. The concept of a “symbiome’ spans plasmids, genetic islands, as well as the rest of the chromosome. It is loosely based on the concept of ‘core’ and ‘accessory’ genomes, where the ‘accessory’ genome has a lower GC content and usually, but not always, is composed of plasmids and/or genomic islands [21].

In general, nodulation gene clusters have been found in close proximity to *nif* and *fix* genes. In rhizobial species carrying plasmids, nodulation *(nod, nol and noe)*, *nif* and *fix*, as well as many secretion related genes, are found in a symbiotic plasmid, while in species or strains without plasmids, the genes are located in laterally transferrable genomic islands, also denominated as symbiotic islands. For example, in *R. leguminosarum* bv. viciae 3841 most symbiotic genes are located in plasmid pRL10 [21], while in *mlo a* 500 kb genomic island carries the genes responsible for symbiosis [11,44]. However, there are exceptions, e.g., *Sinorhizobium* NGR 234, in which *nif* and *nod* operons are located on a plasmid, while the *fix* genes are on the chromosome [34]. Also in *R. etli* CFN 42, the pSym p42d contains most of the genes needed for symbiosis, but homologs for nodulation genes are found in other replicons of the genome [16]. These symbiotic regions of nitrogen fixing *Rhizobiales* genomes have been found to be largely mosaic structures that have been frequently tailored by recombination, horizontal transfer and transposition [15].

In the thirteen symbiotic nitrogen fixing bacterial genomes compared, there is a range of symbiosis region arrangements. The simplest is in *azc*, which contains a small 87.1 kb symbiotic island with *nod* and *trb* operons responsible for the production of Nod factor and type IV secretion, respectively [7]. The *nif* and *fix* gene families are in separate regions of the chromosome. As *azc* is unique amongst the fourteen genomes in being able to fix nitrogen without symbiosis, it has been hypothesised that this symbiotic island has been laterally transferred to *azc* some time after its initial evolution [7].

Both *bja* and *mlo* possess large symbiotic islands (~500 kb) containing all nodulation, *nif* and *fix* genes as well as some secretion related genes [9,44]. The other ten symbiotic nitrogen fixers all have symbiotic plasmids in which most of the nodulation, *nif*, *fix* and secretion genes are located [16,17,21,26,28,45].

#### 2.4.1. *Nod* Genes

Induction of nodulation genes leads to the production and secretion of return signals, the Nod Factors, which are lipochitooligosaccharides of variable structure. Nod factors are essential for the *Rhizobiales* to trigger root hair curling, to induce the formation of nodule primordia, and to enter the root via infection threads. Purified Nod factors are sufficient to induce root hair deformations, cortical cell divisions, and on some host plants, fully grown nodule-like structures. NodD is the core signaling protein, reacting to plant flavonoids then binding to *nod* boxes, binding sites upstream of *nod* genes, typically *nodA* and/or *nodB*, triggering the expression of a *nod* gene cascade and thus the construction of the Nod Factor (e.g., [46]).

The results of BlastlineMCL clustering were utilized to the extraction of all *nod* protein ortholog clusters (Table S3). In total, twenty five different Nod protein ortholog clusters amongst the fourteen species were found. All fourteen species contained *nodD*, *nodE*, *nodG*, *nodI*, *nodM*, *nodP*, *nodQ*, *nodV* and *nodW*. Unsurprisingly, the non-symbiotic *mes* had the least number of *nod* genes, with ten. In a previous study, *nodD* and *nodM* have also been indicated as common genes of bacteria of the order *Rhizobiales*, but including both symbiotic and pathogenic stains [5]. 

One of the Nod orthologs found in all species is NodG, which is in the second largest MCL cluster. It is a cation protein exporter, involved in the secretion of the finished Nod factor into the environment. It is noteworthy that in *R. tropici* strain PRF 81 *nodG* can be promptly transcribed—after only 5 min—in the presence of host specific flavonoids [47]. 

Two *nod* genes were found exclusively in one strain: *nodR* in *R. leguminosarum* bv. *trifolii* WSM1325 and *nodO* in *R. leguminosarum* bv. *viciae* 3804. The function of *nodR* is unknown; however, it is suspected that NodR might contribute to the superior host nodulation efficiency of WSM1325, compared to WSM2304 [20]. In relation to NodO, it catalyzes the addition of carbamoyl to the Nod factor backbone [48], and it is also a calcium-binding protein that promotes infection thread development in root hairs [49]. Interestingly, in *Rhizobium* sp. BR 816 (reclassified as *Sinorhizobium*) the transfer of *nodO* can extend its host range [50], therefore it might be involved in the ability of *rle* to nodulate a wide range of species within the *Viciae* tribe.

While all species have many core and non-core *nod* genes, an investigation of *nod* operon/gene structure in the thirteen symbiotic genomes displays a wide variety of arrangements. The *Sesbania* nodulator *azc* has a large *nod* operon nod*ABCSUIJZ*noe*CHOP* with *nodD1* located downstream [7]. The soybean nodulator *bja* also has a large *nod* operon *nodD1-YABCSUIJ* [9]. Surprisingly, the other two *Bradyrhizobium,* *bbt and bra,* have no *nodA*, *nodB* or *nodC* genes [11], and the signaling mechanisms with the host plant are still under investigation [10]. 

The three *R. leguminosarum* and the two *Sinorhizobium* genomes studied have similar initial *nod* operon structures, *nodDABCIJ*. In *R etli*, the arrangement differs with *nodD*, *nodIJ*, a 200 bp gap and then *nodCB*; the gene *nodA* is 16 kb downstream. Finally, in *mlo*, there is a separate *nodAC* and *nodIJ* operon, with *nodD* and *nodB* in disparate regions of the 600 kb symbiotic island. 

**Figure 3:**
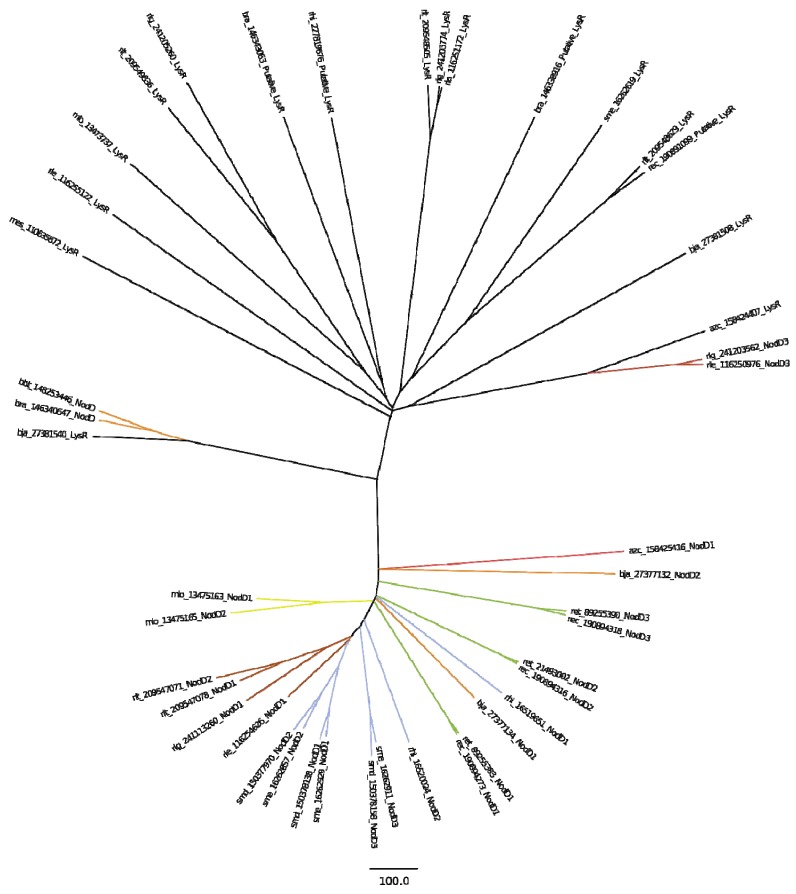
Phylogeny of NodD proteins of the *Rhizobiales* genomes from this study, achieved through a neighbor joining (NJ) gene tree based on BLOSUM-62 matrix alignment of the proteins in the NodD cluster. Number in sequence label is the GENBANK protein GI number. Colored line indicates genus of each genome as coded for in Tables S1 to S18, as well as that the protein is a confirmed NodD protein. Black lines indicate protein is only a putative protein within the LysR family.

The most intriguing organization is found in *rhi*, apparently a composite arrangement. It has a *nodABC* operon, a gap of 152 bp, following a *nodIJ* operon. *nodD1* is ~200 kb downstream from *nodAB* [34]. This arrangement of key *nod* genes in *rhi* seems to be a composite of *R etli,* *R. leguminosarum* and *Sinorhizobium nod* gene arrangements.

The taxonomy of *nod* genes of the fourteen genomes was further explored by measuring the similarity of the NodD proteins within the designated MCL cluster. This was achieved through a neighbor joining (NJ) gene tree based on BLOSUM-62 matrix alignment of the proteins in that cluster (Figure 3). The cluster itself is a broad collection of LysR transcription regulator-like proteins, and it is divided onto two broad branches representing proteins annotated as NodD (colored according to cluster table color scheme) and those annotated only as LysR or putatively LysR (black nodes). This initial division shows that *bra* and *bbt* *nodD1* are possibly not true NodD1 proteins but more general LysR, probably regulating the transcription of other gene systems, e.g., secretion, as both strains have no *nodABC* genes.

Further exploration of the NodD tree shows that the *azc* is the most distant in terms of amino acid domain composition with its closest neighbor being *bja* NodD2. The rest of the proteins in the cluster annotated as Nod largely cluster according to previously determined taxonomic genus relationship, except for *rhi* NodD1, which more closely aligns with *bja* NodD1, rather than with its fellow *Sinorhizobium* species. The largest cluster consists of the NodD1 and NodD2 of *sme* and *smd* with the NodD1 of *rle, rlg* and *rlg* and NodD2 of *rlt*. (Figure 3). 

In summary, there is a significant difference within this protein cluster between nodulation specific LysR type proteins (NodD proteins) and the other LysR transcription regulation orthologs, indicative of their biochemical roles. Also, NodD orthologs show a slightly different taxonomic relationship compared to 16S rRNA and whole genome approaches. 

#### 2.4.2. Other Nodulation Genes

The complexity of Nod factor synthesis and regulation is represented in the current study by two other important gene families, *nol* and *noe*. Among important proteins within this class we may cite NoeI, that catalyses the addition of -*O*-Me fucose to the Nod Factor backbone; NoeE, 3-O-SO3– fucose; NoeL; 3-or 4-*O*-acetyl fucose and NoeC, involved in the addition of arabinose to the Nod factor [48]. *nol* gene family is largely predominant in *bja*, but also abundant in both strains of *R. etli* and in S. *fredii* NGR 234 (Table S5), and thus possibly important for the synthesis of specific Nod factors in these species. 

Many differences were found in *noe* clusters (Table S4). Again, the greatest number of genes in this category was found in *bja*. *Sinorhizobium* species have only *noeA* and *noeB,* and these genes were thought to be specific for *Medicago* nodulation [50]; however, a *noeA* ortholog is found in the *Trifolium* nodulator *rlg* as well as the non-symbiont *mes.* While the functionality of these specific orthologs is not known, this suggests that noeA may not be specific to *Medicago*. 

Protein NolG is ubiquitous, found in cluster 23 and present in all strains from this study. However, it is only annotated as such in *sme*, with *nolG-*like genes annotated in *smd* and *rec*. Similar to the observations in relation to NodG, the NolG cluster contains many proteins, 130, all belonging to the very broad COG ortholog “COG0841, AcrB, cation/multidrug efflux pump”. Therefore it is possible that several orthologs represent only non-nodulation cation pumps. 

#### 2.4.3. Bacterial Secretion Systems

After the initial signaling between the host plant and the rhizobia, the next stage controls the progression of the bacteria into the plant and their maturation into nitrogen fixing bacteroids, and many gene families are related to these steps; among the most crucial genes in these stages are the protein secretion systems. Bacterial secretion systems have been classified into seven main groups, Types I through VII, with the fimbrial chaperone-usher pathway forming an additional group. Four of these systems, Types III, IV, V, and VI, assemble surface structures that contact target cells and deliver DNA and/or protein effectors.

The secretion systems found in *Rhizobiales* range from Type I to Type VI, in addition to the twin-arginine transfer system (Tat). Noteworthy is that different nitrogen fixing *Rhizobiales* with differing host relationships contain different subsets of these systems [34,51,52,53,54]. Previously, it has been reported that only *rhi* had a suite of secretion systems spanning from Type I–V, which may strongly affect its extraordinary host range [34].

Using BlastlineMCL protein clustering and the KEGG pathway orthology, the number of Type I, II, II, IV, V and VI secretion genes, as well as of exopolysaccharide gene families *exo* and *pss* were investigated (Table S7–S14). Interesting, the non-symbiotic *mes* contains many of these secretion systems, including the virulence related Type IV genes. This could be construed as additional evidence that this strain, or a recent ancestor, was once a symbiont, but lost the symbiotic ability due to the availability of alternate energy sources.

##### 2.4.3.1. Tat, Type I and II Secretion Systems

Tat, Type I and Type II secretion systems are the most simple bacterial secretion systems, excreting proteins into extracellular space without the need of host contact. The twin arginine transport (Tat) system is responsible for transporting pre-folded proteins to the periplasmic space. The Tat pathway has been implicated in many bacterial cellular functions, including motility, biofilm formation, pathogenesis and symbiosis [55]. Protein clusters containing TatA, TatB and TatC were found in all fourteen species (Table S6). For TatA, the basic taxonomic pattern was found with TatA clusters separated roughly according to genera. In contrast, TatB clusters were split between the *Azorhizobium*/*Bradyrhizobium* and the other bacteria in a separate cluster, while all TatC proteins fit in one cluster. Therefore, while every species has a Tat system, each one is at least genus specific.

The Type I system is simple and comprises three proteins responsible for the transport of targeted proteins across both bacterial membranes to the extracellular space. This includes ArpD/E, TolC, and the HlyD and HlyB families. In addition, multiple PrtD and PrtE type I proteins have been isolated in *rle*. As shown in Table S7, ArpD/E, HlyB and PrtDE system proteins are found in the one cluster spanning all fourteen species. Type I secretion is generally common across the fourteen *Rhizobiales* genomes, but with a notable absence of TolC in *rec, rlg, rle* and *rlt* and the lack of KEGG Type I secretion proteins orthologs in *mes.*

Type II secretion (*gsp*) was found in *Bradyrhizobium, M. loti* and *Sinorhizobium* NGR 234, and absent in the other genomes (Table S9). The other Type II secretion family, *sec*, was found in all genomes (Table S8). Interesting was the divergence in SecE (preprotein translocase subunit), with twelve different clusters; only *rhi*, *sme* and *smd* secE were positioned in a cluster, while each one of the other genomes occupied a unique cluster. Another contrast was the absence of SecG (preprotein translocase subunit) in *rhi*, *sme* and *smd,* indicating that *Sinorhizobium* may not contain a preprotein translocase composed of three units.

##### 2.4.3.2. Tat, Type III and IV Secretion Systems

Probably the most important secretion systems in regards to the symbiotic relationship are the host contact dependant Type III and Type IV secretion systems [48,54,56]. Type III secretion system is responsible for producing nodulation outer proteins (Nops), some of which may be delivered into host plant cells via pili on the bacterial surface, and are also closely involved in flagella systems [57]. These Type III systems are present in the KEGG orthology as Ysc. Many species are annotated with different names such as *fli* and *hrc*. Amongst the fourteen genomes, Type III protein orthologs are present in *bja, mlo, ret, rec* and *rhi*, and absent in *rlg, rlt, rle, smd* and *sme* (Table S10). One might assume from these results that the presence of Type III in *Sinorhizobium* NGR 234 could be implicated in its ability to nodulate the hosts of *bja, mlo, ret* and *rec.*

In the *Rhizobiales*, Type IV secretion proteins are found in two broad families, F-type (Table S11), which includes the *virB* and *trb* families, and the P-type *cpa/tab/pli* system (Table S12). The *virB/trb* genes are tightly related to virulence and conjugal transfer, while P-type are thought to be adapted from flagella proteins. The *trb* system was first described in *Agrobacterium* Ti plasmid as coding for the key virulence system. In the genomes from this study, *azc*, *brj*, *bbt*, *mlo* and *mes* contain these genes. The other *Rhizobiales* have *virB* orthologs in differing clusters from most of the *trb*, thus opposed from the *A. tumefaciens* model. Nonetheless, F-type IV seems to be ubiquitous across the fourteen genomes, including *mes,* with many of the species having multiple paralogs of many of the *virB* genes. A similar situation is observed with the P-type secretion mechanism that is found all but *azc* genome, a further indicator of its relatively recent evolution in nodulation ability.

##### 2.4.3.3. Type V and Type VI Secretion Systems

Type V (auto-transporters) secretion system possesses the simplest secretion apparatus and represents the largest family of protein translocating outer membrane porins in Gram-negative bacteria [58]. Paradoxically, it is not common in the rhizobial genomes studied. Type V is represented in all fourteen gemomes by three orthologs, *autA*, *autB* and *autC* (Table S13), first isolated in *rle* [21], with *mes* being the only other genome under study that contained orthologs for all three Aut proteins. The existence of Type V orthologs is further evidence that *mes,* despite being non-symbiotic, still has virulence or infection potential. 

Type VI is a newly discovered system based on bacteriophage secretion systems. First described in 2006, Type VI system has been found in many bacteria, with possible orthologs in *mlo*, *bja* and *rle* [59,60]. A scan of the BlastlineMCL clusters, based on the previous *mlo*, *bja* and *rle* findings [59,60], found possible orthologs also in *azc* and *rec* (Table S14). Noteworthy is the fact that *rle* is the only nitrogen fixer with full sets of Type V and Type VI secretion systems. As both *rle* and *rhi* nodulate members of the *Viciae* tribe, that might suggest some differences in the secretion and communication between microsymbionts and *Viciae*, and points out that the role of Type V and Type VI secretion systems represent an intriguing question to be explored.

#### 2.4.4. Exopolysaccharide Production

Exopolysaccharide production has shown to be critical in host symbiont relationships, e.g., for the initial bacterial invasion that leads to the formation of indeterminate type of nodules on legumes [61]. The examination of exopolysaccharide synthesis and its role in legume infection has been mainly focused on the *S. meliloti—Medicago truncatula* model, with *sme* producing two exopolysaccharides, EPS I (succinoglycan) and EPS II (galactoglucan) [46]. In our comparison of genomes two exopolysaccharide synthesis families were surveyed, related to *exo* and *pss* genes; these were originally described in *S. meliloti* and *R. leguminosarurm,* respectively, and produce alternatively structured EPS I.

In a few cases, the ontology of exopolysaccharide synthesis proteins in nitrogen fixing *Rhizobiales* seems to be overlapping with four *exo* and *pss* terms used interchangeably [21]. For example, *exoM* is clustered with *pssC* in the *R. etli* species, both coding for a glycosyltransferase. This could be either an ontology clash, that the two proteins are in the same cluster due to similar amino acid structure, or that the same single protein is involved in the two systems. 

Overall the clustering of *exo* (Table S15), and *pss* (Table S16) was similar to the phylogenetic clustering The African nodulator *azc* has only seven *exo* and three *pss* orthologs, compared to the twenty-three and six orthologs, respectively, found in *sme.* In addition, *Bradyrhizobium* has also few *exo* and *pps* genes, while *Mesorhizobium* has few *pps genes.* This interesting comparison could be indicative of a limited EPS I production in these microsymbionts, or that they use different strategies or sets of genes. It cannot be related to the tropical origin or the formation of determinate nodules, as other rhizobia, e.g., the tropical *R. etli* has several EPS genes and forms determinate nodules in common beans. Still considering the *pps* family, both *ret* and *rle* have twenty *pss* orthologs. Furthermore, EPS may also play other important roles, such as providing environmental protection, as pointed out in proteomic and transcriptomic studies with *B. japonicum* [62,63]. Continuing, the *Trifolium* nodulators have eighteen *pss* orthologs, missing *pssH* and *pssI*. Finally, *rec* has sixteen *pps*, missing *ppsF*, *pssH*, *pssI*, *pssJ* and *pssK*. In the other genomes the number of *pps* orthologs range from only three in *azc* to six in *Sinorhizobium*. 

The role and importance of EPS in the symbiosis has been long discussed and studied. However, the comparison of the genomes has shown that the complexity of the genes regulating EPS is far from being understood. Different species have adopted different sets of genes that seem more related to evolution of the core genome than of the symbiotic genes. Therefore, it can be concluded that the production of exopolysaccharide is not necessarily a feature of a ‘universal’ symbiome.

#### 2.4.5. *Nif* and *Fix* Genes

The fixation of nitrogen *via* nitrogenase requires an anaerobic or micro-aerobic bacteroid environment and the *fix* gene family is involved in the regulation and metabolism of oxygen in this circumstance. The *fix* gene family is commonly found in three core operon structures: *fixABCX*, *fixGHIS* and *fixNOPQ*. The first operon is involved in the regulation of gene transcription under low oxygen concentrations. Metabolism of oxygen by the bacteroid occurs via the cytochrome *cbb3* oxidase complex, a membrane complex encoded by the *fixNOPQ* operon, that mediates electron exchange and synthesis of ATP via oxidation on the outer surface of the bacteroid membrane. Studies in *bja* have shown that the second operon, *fixGHIS*, is required for the initial construction of the *cbb3* complex, and like *fixNOPQ*, is only expressed under micro-aerobic or anaerobic conditions.

BlastlineMCL clustering of the Fix proteins shows both important species distinctions as well as a possible limitation to the algorithm (Table S17). All thirteen symbiotic species have the complete set of genes of the three operons; in the non-symbiont *mes*, *fixABCX* is entirely missing, as well as *fixQ*. 

Each different species, except for *azc*, has a unique *fixS* ortholog cluster, and *mlo* has two. However, as the FixS protein is only fifty five amino acids in size, this could also be related to a limitation of the algorithm used by BlastineMCL, adjusted to larger proteins. Another stem nodulator, *bbt,* has no *fixQ* ortholog, signifying different *cbb3* apparatuses from the structural models already resolved. This diversity of both FixQ and FixS proteins may suggest a diverse range of *cbb3* complexes for each species that could result from differences in host nodulation strategies.

The *nif* family codes for the MoFe-dependant nitrogenase complex, the enzyme required for the catalysis for the nitrogen fixing process [64]. The thirteen symbionts contained, with a higher or lower similarity, the *nifBHDKENX* operon, which in turn was absent in the non-fixer *mes* (Table S18). Therefore, along with *nod* and *fix*, this species has also lost *nif* genes.

While a lack of *nifX* has been previous reported in *rle* [65], the current analysis has shown that both *nifX* and *nifZ* are also absent in *R. leguminosarum* *rlt* and *rlg*. This suggests the MoFe nitrogenase in these *leguminosarum* species may have a distinct structure from the other *Rhizobiales*. Also confirmed is the lack of *nifQ*, required for Mo-incorporation into the complex, in *R*. *leguminosarum*, *S. meliloti* and *S. medicae*, as well as in the non-symbiotic *mes*. Consequently, these species should use other means to incorporate Mo into the nitrogenise complex [65,66].

**Figure 4:**
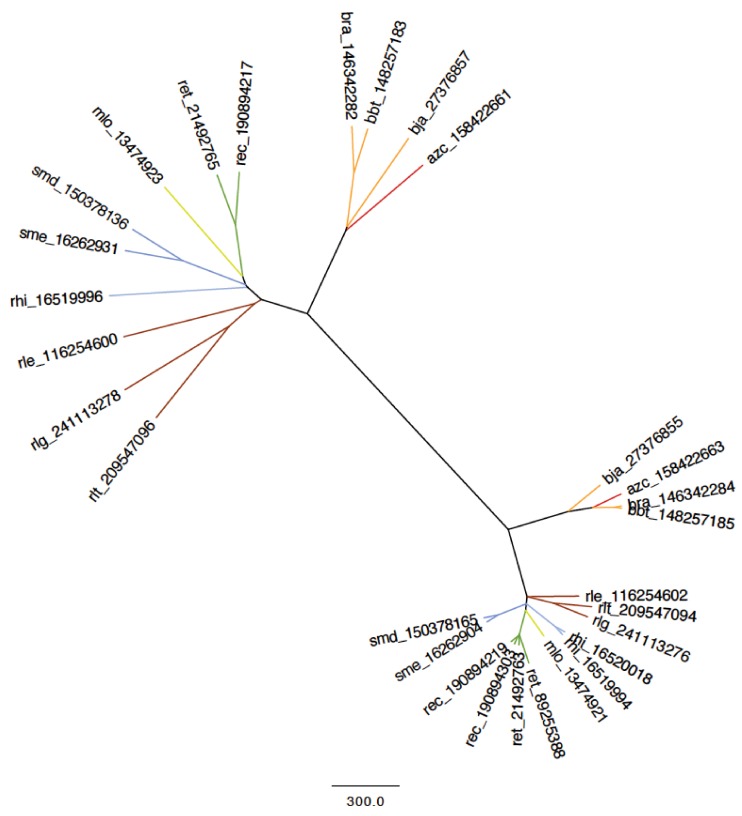
Phylogeny of NifN/K proteins of the *Rhizobiales* genomes from this study, achieved through a neighbor joining (NJ) gene tree based on BLOSUM-62 matrix alignment of the proteins in the NifN/K cluster. Number in sequence label is the GENBANK protein GI number. Colored line indicates genus of each genome as coded for in Tables S1 to S18.

A Nif ortholog cluster was chosen for more in depth analysis of protein alignment (Figure 4). In this example, the NifN/K cluster was examined via neighbor joining (NJ) tree based on BLOSUM62 matrix. Both NifN and NifK (considered structural homologs in regards to their role in nitrogenase complex) split into two clear clades. Unlike much of the NodD cluster, the NifN clade is in agreement with the 16S rRNA phylogeny. Interestingly, the *Bradyrhizobium* and *Azorhizobium* NifN orthologs are genetically very distant from the other species, suggesting a different nitrogenase structure. A lower agreement with the 16S rRNA was obtained in the analysis of NifK; more specifically, NifK homologs of *rhi* show low similarity with other *Sinorhizobium* NifK homologs, showing higher similarity with *mlo* and with the *R. leguminosarum* orthologs. This is another suggestion of an important event of lateral transfer.

### 2.5. Is it Possible to Define a Symbiome?

As mentioned previously, the definition of a prokaryotic genome is rapidly changing as the number of genomes sequenced and annotated rises. The field of metagenomics has especially caused researchers to re-assess the nature of prokaryotic taxonomy and evolution, for example as a disconnected network topology rather than an exclusively cell and tree centered taxonomy [67]. This is arguably the case for the concept of a “symbiome”. However, the results of this study raise doubts about the definition of a universal “symbiome”: clearly, there are vastly different structures and profound differences in the set of symbiotic regions and genes spanning both chromosomes and plasmids.

The sharply different chromosome and plasmid symmetry taxonomies obtained in the comparison of the fourteen genomes have highlighted a disconnected network pattern caused by lateral transfer within the order *Rhizobiales*. While chromosome symmetry strongly correlates with genus and species designation, plasmid symmetry is much weaker, especially amongst symbiotic plasmids. This could be construed as evidence of multiple lateral transfer events and rearrangements of core symbiotic genes compared to lesser transfer on other non-symbiotic genes. A good example relies in the symbiotic plasmid of *Sinorhizobium* NGR 234, that lacks symmetry (e.g., considering the critical protein NodD, Figure 3) with all the other symbiotic plasmid and genome islands analyzed. In addition, *rhi* has apparently non-*Sinorhizobium* NifK and NifN proteins (Figure 4). As NGR 234 can nodulate a broad range of legumes, this might suggests a unique re-arrangement of nodulation genes to allow the highest symbiotic ubiquity reported so far.

Protein cluster analysis has shown a much more complex pattern of correlation with host range and protein content, an example being the large array of secretion mechanisms available in the fourteen genomes. Some examples are that the genomes of *bja*, *mlo* and *rhi* contain the majority of Type II secretion proteins, while *rle* and *ret* do not contain any. Type III orthologs are found in *rec*, *ret* and *rhi*, but not in *R. leguminosarum* species, or in the other two *Sinorhizobium*. All species, except for *azc* and *bra*, contain most of the orthologs of Type IV. Intriguingly, *rle* is the only species with a full complement of both Type V and Type VI secretion orthologs. While NGR 234 (*rhi*) lacks these Type V and VI secretion proteins, as well as *pss* exopolysaccharide production enzymes, it can still successfully nodulate both *Viciae* and *Trifollium* species [32]. In addition, NGR contains a large number of Type II and III secretion orthologs, indicative of its ability to nodulate the hosts of *Bradyrhizobium* and *Mesorhizobium* [32,34]. Interesting, there was an overall lack of secretion genes in *azc* compared to the other species. One interpretation is that this omission could be related to the capacity of the species to fix nitrogen in free living conditions and only gaining the symbiotic ability in a later stage of its evolution [7]. The non-nitrogen fixing, non symbiont *mes* demonstrates a reverse situation, evolving from a symbiotic nitrogen fixing ancestor to a chemotroph, with the lack of a symbiotic island *i.e.*, no *nif*, *nodDABC* or *fixABCX* orthologs, but still retaining a wide range of other *nod*, *fix* as well as secretion and exopolysaccharide system orthologs typical of symbiotic nitrogen fixing organisms. Altogether, these differences among the fourteen rhizobial genomes highlight the difficulties of defining a “symbiome”.

## 3. Experimental Section

### 3.1. Genomes

All fourteen *Rhizobiales* genomes, including chromosomes and, if applicable, plasmids, were downloaded from NCBI RefSeq. These include three genome sequenced and analyzed by the Center for *Rhizobium* Studies with the suffix WSM (Western Australian Soil Microbiology Collection). All available nucleotide and protein FASTA files, as well as GENBANK files were utilized. See Table S1 for details.

### 3.2. Bioinformatics Analysis

Taxonomic analysis of the 16s rRNA gene sequences was completed with MEGA4 [68], by constructing a bootstrapped neighbor joining (NJ) gene tree using Jukes-Cantor substitution. Dotplots (http://code.google.com/p/freckle/) were constructed from the above chromosomal and plasmid nucleotide FASTA files using an in house script. The BLASTMATRIX of all protein sequences and extraction and analysis of KEGG data were performed as outlined in Bellgard *et al.* 2009 [69].

Construction of phylogenetic NJ trees was based on a CLUSTALW alignment with BLOSUM62 matrix, all accomplished with JALVIEW 2.6.1 [70]. Further editing of trees to publication standard was completed with FigTree v1.3.1 (http://tree.bio.ed.ac.uk/software/figtree/). 

### 3.3. Protein Clustering

A protein reciprocal Blast similarity search with a threshold maximum expected value 1e^−20^ was conducted. This protein clustering used BlastlineMCL (http://www.micans.org/mcl/) program that implements the MCL cluster algorithm for graphs. The granularity of the output cluster was set with an inflation value of 2.5. A bioinformatics workflow was developed that would for each cluster, annotate the cluster based on domain conservation (NCBI CDD), produce a cluster multiple sequence alignment and phylogenic tree for web publication. The clustering analysis can be viewed at the following web site: http://ccg.murdoch.edu.au/organism/rhizobium/RhizobiumSummaries/

The workflow is freely available for researchers interested in high-through put clustering and annotation by contacting mbellgard@ccg.murdoch.edu.au (reference IVEC and CCG).

## 4. Concluding Remarks

Overall, it can then be concluded from this study that protein clustering has demonstrated that there are multiple overlapping strategies for nitrogen fixing symbiosis and that perhaps the concept of a single self contained ‘symbiome’ is doubtful. Expanding this protein cluster comparison from *Rhizobiales* genomes to a larger range of nitrogen fixers could further resolve the symbiome conundrum. To date several species that do not belong to order *Rhizobiales* but establish nitrogen fixing symbioses have been discovered. These include several species of the beta-proteobacteria *Burkholderia* [71] and *Cupriavidus taiwanensis* [72] as well as the non-legume root nodule symbiosis with the Gram positive *Actinomycetales* genus *Frankia* [73]. Therefore, a wider exploration of several classes of bacteria would have to be considered. 

This wider approach is demonstrated, in part, by Pini *et al.* 2011 (92 alphaproteobacterial genomes) [74] and Amadou *et al.* 2008 (1 betaproteobacterial and 8 alphaproteobacterial genomes) [72]. These studies utilized significantly different clustering techniques and did not include all the 14 strains explored in the current study. However, both these studies share our view, that while there is a large shared gene pool amongst the plant symbionts, there are “multiple recipes” [74] possibly due to the different host environments [72]. This adds further doubt to the concept of a single self-contained “symbiome”. 

One must be conscious of the fact that just predicting an ortholog does not guarantee that the said ortholog is actually expressed. Therefore, despite the number of genomes available to be compared, a wider systems biology approach should also be taken into account. This would include symbiont transcriptomics, protein-protein interactions, the effect of the presence of other prokaryotic and eukaryotic species in the rhizosphere, and the genomics and transcriptomics of the plant host itself. Many pertinent studies have already been completed, such as the construction of a *S. meliloti* interactome [75], the demonstration of host dependant gene expression, based on extensive proteomic and transciptomic analysis in *bja* [76] and in *azc* [8], the role of mychorrhizal fungi in the development of nodules [77], and the increasingly complex role of plant nodule-specific cysteine-rich peptides on the symbiotic relationship between legume and bacterium [78], such as host control of *R. leguminosarum* bacteroid formation through symbiotic auxotrophy [79]. Therefore, if there were any nitrogen fixing ‘symbiome’, it would more likely to be a complex composition of molecular biological and biochemical networks from both symbiont and host in genomics, transcriptomics, proteomics, and even metabolomics. Further, comparative genomics and proteomics, as shown in this study, represents a valuable and key tool for capturing specificities and generalities of each genome, or of groups of genomes showing relevant functionalities, as is the case of nitrogen fixing symbiotic bacteria.

## Supplementary Files

Supplementary File 1 DOCX-Document (DOCX, 685 KB)
