# Supplementary material for: The Genetics of Symbiotic Nitrogen Fixation: Comparative Genomics of 14 Rhizobia Strains by Resolution of Protein Clusters
Source: Genes (Basel). 2012 Feb 16;3(1):138–66. doi: 10.3390/genes3010138 (PMC3899959; doi:10.3390/genes3010138)
Supplement: Supplementary File 1 — DOCX-Document (DOCX, 685 KB) [file genes-03-00138-s001.docx]

**Supplementary Files**

**Figure S1.** FRECKLE DNA dotplot of the fourteen *Rhizobiales* plasmids, constructed from nucleotide FASTA files using an in house script.

**
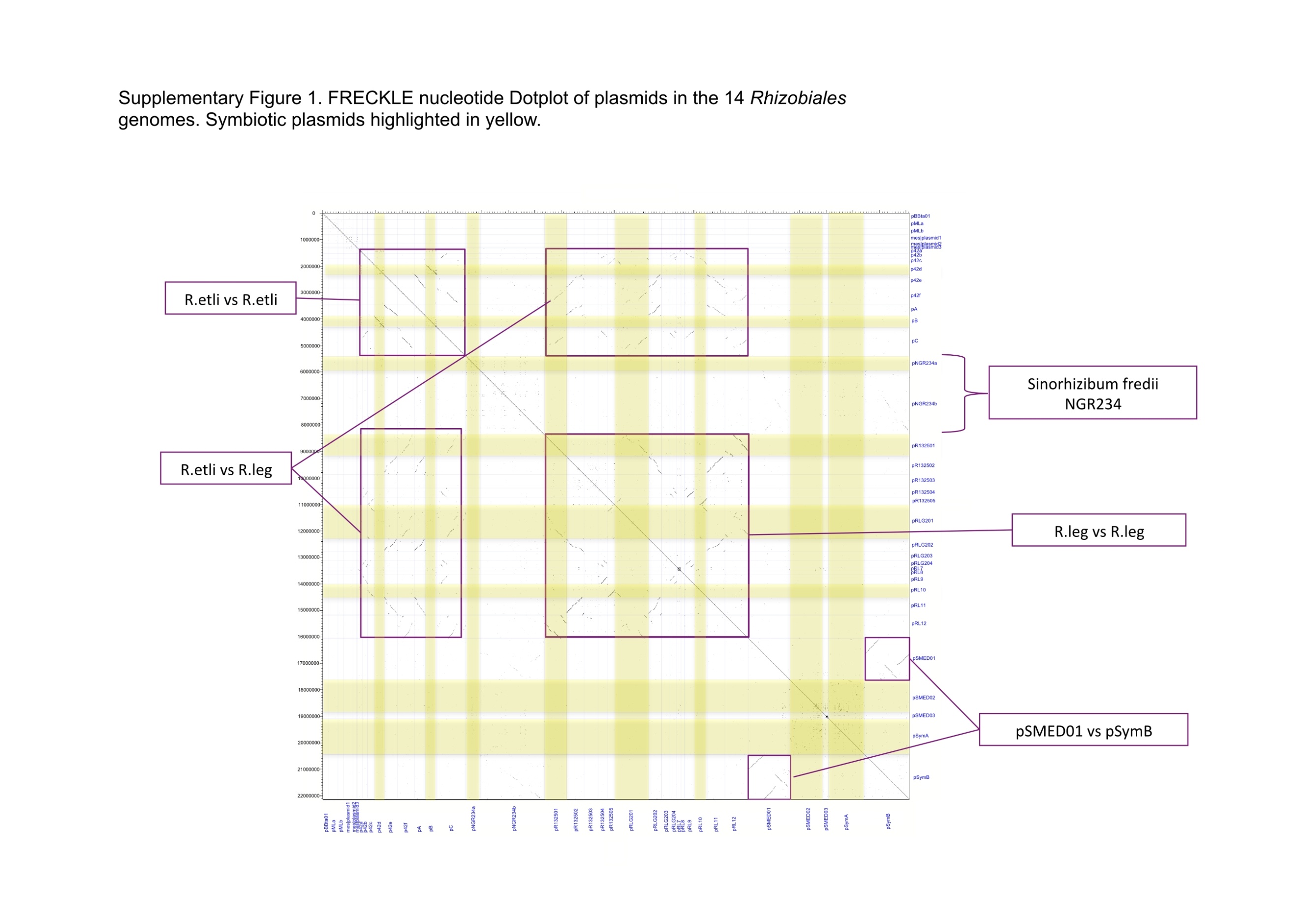
**

**Tables S1 to S18**—Assorted gene clusters from blastlinemcl analysis.

**Table S1.** Full table of KEGG pathways of the fourteen genomes.

| **Relevant KEGG Pathways** | **KEGG ORTHOLOGS** | | | | | | | | | | | | | |
| --- | --- | --- | --- | --- | --- | --- | --- | --- | --- | --- | --- | --- | --- | --- |
|  | **azc** | **bja** | **bbt** | **bra** | **mlo** | **mes** | **ret** | **rec** | **rlg** | **rlt** | **rle** | **rhi** | **smd** | **sme** |
| **1. Metabolism** | **1048** | **1253** | **1357** | **1338** | **1225** | **1036** | **1079** | **1253** | **1052** | **1072** | **1315** | **1079** | **1079** | **1230** |
| **1.1 Carbohydrate Metabolism** | **240** | **295** | **295** | **296** | **274** | **245** | **249** | **279** | **257** | **256** | **299** | **262** | **267** | **273** |
| ko00010 Glycolysis/Gluconeogenesis | 21 | 26 | 27 | 25 | 25 | 22 | 20 | 22 | 20 | 20 | 24 | 22 | 24 | 26 |
| ko00020 Citrate cycle (TCA cycle) | 21 | 24 | 23 | 23 | 23 | 22 | 22 | 21 | 22 | 23 | 22 | 22 | 21 | 21 |
| ko00620 Pyruvate metabolism | 33 | 33 | 34 | 32 | 30 | 31 | 29 | 30 | 27 | 28 | 33 | 26 | 30 | 28 |
| ko00030 Pentose phosphate pathway | 19 | 25 | 25 | 24 | 25 | 25 | 22 | 22 | 23 | 23 | 24 | 26 | 25 | 25 |
| ko00040 Pentose and glucuronate interconversions | 13 | 13 | 12 | 10 | 13 | 11 | 13 | 18 | 15 | 13 | 20 | 13 | 15 | 17 |
| ko00051 Fructose and mannose metabolism | 8 | 17 | 18 | 18 | 20 | 10 | 8 | 15 | 9 | 8 | 16 | 12 | 10 | 15 |
| ko00520 Amino sugar and nucleotide sugar metabolism | 19 | 25 | 24 | 27 | 24 | 22 | 22 | 27 | 24 | 24 | 28 | 25 | 23 | 28 |
| ko00052 Galactose metabolism | 6 | 10 | 8 | 10 | 12 | 10 | 12 | 14 | 14 | 13 | 14 | 11 | 10 | 13 |
| ko00500 Starch and sucrose metabolism | 12 | 18 | 19 | 19 | 14 | 13 | 19 | 23 | 20 | 21 | 22 | 19 | 16 | 5 |
| ko00053 Ascorbate and aldarate metabolism | 6 | 7 | 7 | 7 | 4 | 5 | 5 | 7 | 6 | 5 | 6 | 6 | 7 | 6 |
| ko00630 Glyoxylate and dicarboxylate metabolism | 26 | 30 | 26 | 29 | 22 | 21 | 21 | 22 | 22 | 22 | 26 | 23 | 25 | 26 |
| ko00640 Propanoate metabolism | 23 | 26 | 30 | 27 | 22 | 20 | 20 | 21 | 20 | 21 | 26 | 20 | 24 | 22 |
| ko00562 Inositol phosphate metabolism | 4 | 4 | 7 | 8 | 8 | 6 | 9 | 9 | 8 | 8 | 9 | 8 | 9 | 9 |
| ko00650 Butanoate metabolism | 22 | 31 | 28 | 30 | 25 | 21 | 21 | 22 | 21 | 21 | 23 | 23 | 22 | 26 |
| ko00660 C5-Branched dibasic acid metabolism | 7 | 6 | 7 | 7 | 7 | 6 | 6 | 6 | 6 | 6 | 6 | 6 | 6 | 6 |
| **1.2 Energy Metabolism** | **114** | **147** | **146** | **145** | **113** | **114** | **112** | **111** | **99** | **99** | **118** | **111** | **124** | **138** |
| ko00910 Nitrogen metabolism | 26 | 33 | 30 | 28 | 21 | 17 | 18 | 27 | 18 | 20 | 22 | 27 | 27 | 30 |
| ko00195 Photosynthesis | - | - | 8 | 8 | - | - | - | - | - | - | - | - | - | - |
| ko00190 Oxidative phosphorylation | 52 | 50 | 51 | 52 | 50 | 50 | 47 | 48 | 47 | 47 | 51 | 47 | 47 | 49 |
| ko00680 Methane metabolism | 27 | 17 | 18 | 18 | 16 | 10 | 10 | 12 | 10 | 10 | 16 | 12 | 11 | 16 |
| ko00920 Sulfur metabolism | 9 | 11 | 10 | 11 | 9 | 9 | 8 | 9 | 8 | 8 | 10 | 9 | 9 | 12 |

**Table S1.** *Cont.*

| **Relevant KEGG Pathways** | **KEGG ORTHOLOGS** | | | | | | | | | | | | | |
| --- | --- | --- | --- | --- | --- | --- | --- | --- | --- | --- | --- | --- | --- | --- |
|  | **azc** | **bja** | **bbt** | **bra** | **mlo** | **mes** | **ret** | **rec** | **rlg** | **rlt** | **rle** | **rhi** | **smd** | **sme** |
| **1.2 Energy Metabolism** | **114** | **147** | **146** | **145** | **113** | **114** | **112** | **111** | **99** | **99** | **118** | **111** | **124** | **138** |
| ko00720 Reductive carboxylate cycle (CO2 fixation) | - | 17 | 17 | 17 | - | 13 | 14 | - | - | - | - | - | 12 | 12 |
| ko00710 Carbon fixation in photosynthetic organisms | - | 19 | 20 | 19 | 17 | 15 | 15 | 15 | 16 | 14 | 19 | 16 | 18 | 19 |
| **1.3 Lipid Metabolism** | **52** | **63** | **72** | **72** | **72** | **53** | **61** | **76** | **58** | **57** | **81** | **56** | **58** | **71** |
| ko00061 Fatty acid biosynthesis | 12 | 13 | 13 | 13 | 13 | 13 | 14 | 13 | 13 | 13 | 13 | 14 | 13 | 13 |
| ko00071 Fatty acid metabolism | 11 | 14 | 13 | 13 | 13 | 11 | 9 | 13 | 8 | 8 | 12 | 8 | 9 | 12 |
| ko00072 Synthesis and degradation of ketone bodies | 4 | 6 | 4 | 5 | 3 | 2 | 2 | 2 | 2 | 2 | 3 | 3 | 3 | 3 |
| ko00100 Steroid biosynthesis | - | - | 1 | 1 | - | - | - | - | - | - | - | 1 | - | - |
| ko00120 Primary bile acid biosynthesis | - | 1 | 1 | 1 | 2 | - | - | - | - | - | 2 | - | - | - |
| ko00121 Secondary bile acid biosynthesis | 1 | - | - | - | 1 | - | - | - | - | - | 1 | - | - | - |
| ko00140 Steroid hormone biosynthesis | - | 1 | 2 | - | 3 | - | - | 1 | - | - | 3 | - | - | 2 |
| ko00564 Glycerophospholipid metabolism | 14 | 17 | 19 | 18 | 17 | 15 | 18 | 20 | 17 | 17 | 23 | 14 | 15 | 16 |
| ko00565 Ether lipid metabolism | - | - | 1 | 1 | - | - | - | 1 | - | - | 3 | - | - | - |
| ko00590 Arachidonic acid metabolism | - | 2 | 3 | 3 | 1 | 1 | 2 | 3 | 2 | 2 | 2 | 1 | 1 | 1 |
| ko00600 Sphingolipid metabolism | - | 1 | 1 | 1 | 3 | - | 1 | 2 | 2 | 1 | 4 | 2 | 1 | 4 |
| ko01040 Biosynthesis of unsaturated fatty acids | 3 | 6 | 6 | 5 | 5 | 4 | 5 | 7 | 6 | 6 | 6 | 4 | 5 | 6 |
| ko00591 Linoleic acid metabolism | - | 1 | 1 | 1 | 1 | - | - | 1 | - | - | 1 | - | - | 1 |
| ko00592 alpha-Linolenic acid metabolism | 1 | - | - | - | 1 | 1 | - | 1 | - | - | 1 | - | - | 1 |
| ko00561 Glycerolipid metabolism | 6 | 8 | 7 | 10 | 12 | 6 | 10 | 13 | 10 | 10 | 13 | 9 | 11 | 12 |
| **1.4 Nucleotide Metabolism** | **102** | **96** | **100** | **99** | **111** | **105** | **100** | **108** | **97** | **99** | **112** | **97** | **98** | **114** |
| ko00240 Pyrimidine metabolism | 42 | 39 | 40 | 37 | 44 | 40 | 41 | 44 | 39 | 42 | 44 | 38 | 39 | 44 |
| ko00230 Purine metabolism | 60 | 57 | 60 | 62 | 67 | 65 | 59 | 64 | 58 | 57 | 68 | 59 | 59 | 70 |
| **1.5 Amino Acid Metabolism** | **221** | **249** | **268** | **258** | **275** | **224** | **235** | **261** | **227** | **241** | **272** | **239** | **236** | **253** |
| ko00250 Alanine, aspartate and glutamate metabolism | 21 | 23 | 21 | 20 | 21 | 19 | 22 | 23 | 22 | 23 | 24 | 23 | 22 | 22 |
| ko00260 Glycine, serine and threonine metabolism | 29 | 26 | 32 | 30 | 40 | 29 | 32 | 35 | 30 | 33 | 35 | 33 | 31 | 35 |

**Table S1.** *Cont.*

| **Relevant KEGG Pathways** | **KEGG ORTHOLOGS** | | | | | | | | | | | | | |
| --- | --- | --- | --- | --- | --- | --- | --- | --- | --- | --- | --- | --- | --- | --- |
|  | **azc** | **bja** | **bbt** | **bra** | **mlo** | **mes** | **ret** | **rec** | **rlg** | **rlt** | **rle** | **rhi** | **smd** | **sme** |
| **1.5 Amino Acid Metabolism** | **221** | **249** | **268** | **258** | **275** | **224** | **235** | **261** | **227** | **241** | **272** | **239** | **236** | **253** |
| ko00290 Valine, leucine and isoleucine biosynthesis | 16 | 16 | 16 | 16 | 16 | 16 | 15 | 15 | 15 | 15 | 15 | 15 | 15 | 15 |
| ko00270 Cysteine and methionine metabolism | 16 | 21 | 25 | 21 | 25 | 18 | 19 | 21 | 19 | 18 | 23 | 19 | 22 | 22 |
| ko00280 Valine, leucine and isoleucine degradation | 19 | 23 | 19 | 21 | 20 | 18 | 12 | 19 | 15 | 17 | 20 | 19 | 21 | 21 |
| ko00300 Lysine biosynthesis | 16 | 13 | 14 | 14 | 12 | 12 | 12 | 12 | 11 | 12 | 13 | 12 | 12 | 12 |
| ko00310 Lysine degradation | 9 | 10 | 10 | 10 | 11 | 8 | 10 | 11 | 9 | 10 | 11 | 9 | 9 | 9 |
| ko00330 Arginine and proline metabolism | 30 | 34 | 38 | 35 | 39 | 34 | 40 | 36 | 34 | 36 | 41 | 37 | 38 | 38 |
| ko00340 Histidine metabolism | 13 | 17 | 19 | 18 | 20 | 16 | 16 | 19 | 16 | 16 | 19 | 17 | 15 | 17 |
| ko00350 Tyrosine metabolism | 12 | 19 | 23 | 22 | 17 | 17 | 12 | 21 | 11 | 14 | 18 | 14 | 10 | 15 |
| ko00360 Phenylalanine metabolism | 11 | 14 | 16 | 15 | 17 | 8 | 14 | 15 | 14 | 14 | 17 | 13 | 11 | 14 |
| ko00380 Tryptophan metabolism | 12 | 16 | 16 | 16 | 16 | 10 | 12 | 13 | 12 | 13 | 13 | 11 | 12 | 13 |
| ko00400 Phenylalanine, tyrosine and tryptophan biosynthesis | 17 | 17 | 19 | 20 | 21 | 19 | 19 | 21 | 19 | 20 | 23 | 17 | 18 | 20 |
| **1.6 Metabolism of Other Amino Acids** | **50** | **58** | **62** | **57** | **60** | **48** | **54** | **58** | **54** | **57** | **58** | **56** | **54** | **62** |
| ko00410 beta-Alanine metabolism | 7 | 10 | 13 | 11 | 12 | 8 | 9 | 10 | 9 | 9 | 11 | 11 | 9 | 10 |
| ko00430 Taurine and hypotaurine metabolism | 3 | 5 | 4 | 3 | 4 | 4 | 4 | 4 | 4 | 5 | 3 | 3 | 5 | 7 |
| ko00440 Phosphonate and phosphinate metabolism | 2 | 4 | 4 | 4 | 4 | 2 | 3 | 3 | 3 | 3 | 3 | 2 | 3 | 4 |
| ko00450 Selenoamino acid metabolism | 12 | 12 | 12 | 11 | 15 | 11 | 11 | 14 | 11 | 11 | 12 | 12 | 11 | 15 |
| ko00460 Cyanoamino acid metabolism | 6 | 7 | 6 | 5 | 4 | 4 | 6 | 6 | 6 | 7 | 6 | 5 | 5 | 5 |
| ko00471 D-Glutamine and D-glutamate metabolism | 4 | 4 | 5 | 5 | 4 | 3 | 4 | 4 | 4 | 4 | 5 | 5 | 4 | 4 |
| ko00472 D-Arginine and D-ornithine metabolism | 1 | 1 | 1 | 1 | 1 | 1 | 1 | 1 | 1 | 1 | 1 | 1 | 1 | 1 |
| ko00473 D-Alanine metabolism | 3 | 3 | 3 | 3 | 3 | 3 | 3 | 3 | 3 | 3 | 3 | 3 | 3 | 3 |
| ko00480 Glutathione metabolism | 12 | 12 | 14 | 14 | 13 | 12 | 13 | 13 | 13 | 14 | 14 | 14 | 13 | 13 |
| **1.7 Glycan Biosynthesis and Metabolism** | **30** | **29** | **31** | **33** | **32** | **25** | **33** | **35** | **32** | **34** | **36** | **31** | **22** | **32** |
| ko00510 N-Glycan biosynthesis | - | 1 | 1 | 1 | 1 | - | 1 | 1 | - | 1 | 1 | - | - | - |
| ko00531 Glycosaminoglycan degradation | - | - | - | - | - | - | 1 | 1 | 1 | 1 | 2 | 1 | 1 | 1 |

**Table S1.** *Cont.*

| **Relevant KEGG Pathways** | **KEGG ORTHOLOGS** | | | | | | | | | | | | | |
| --- | --- | --- | --- | --- | --- | --- | --- | --- | --- | --- | --- | --- | --- | --- |
|  | **azc** | **bja** | **bbt** | **bra** | **mlo** | **mes** | **ret** | **rec** | **rlg** | **rlt** | **rle** | **rhi** | **smd** | **sme** |
| **1.7 Glycan Biosynthesis and Metabolism** | **30** | **29** | **31** | **33** | **32** | **25** | **33** | **35** | **32** | **34** | **36** | **31** | **22** | **32** |
| ko00603 Glycosphingolipid biosynthesis—globo series | - | - | - | 1 | 1 | - | 2 | 2 | 2 | 2 | 2 | 2 | 2 | 2 |
| ko00604 Glycosphingolipid biosynthesis—ganglio series | - | - | - | - | - | - | 1 | 1 | 1 | 1 | 1 | 1 | 1 | 1 |
| ko00540 Lipopolysaccharide biosynthesis | 15 | 12 | 14 | 15 | 14 | 9 | 9 | 9 | 9 | 10 | 9 | 9 | - | 9 |
| ko00550 Peptidoglycan biosynthesis | 15 | 16 | 16 | 16 | 16 | 16 | 16 | 16 | 16 | 16 | 16 | 16 | 16 | 16 |
| ko00511 Other glycan degradation | - | - | - | - | - | - | 3 | 5 | 3 | 3 | 5 | 2 | 2 | 3 |
| **1.8 Metabolism of Cofactors and Vitamins** | **117** | **132** | **143** | **137** | **131** | **103** | **122** | **126** | **114** | **117** | **130** | **119** | **117** | **124** |
| ko00730 Thiamine metabolism | 10 | 11 | 11 | 11 | 10 | 9 | 11 | 11 | 8 | 11 | 12 | 9 | 9 | 10 |
| ko00740 Riboflavin metabolism | 9 | 7 | 9 | 9 | 9 | 7 | 7 | 10 | 7 | 7 | 9 | 6 | 7 | 8 |
| ko00750 Vitamin B6 metabolism | 6 | 6 | 5 | 5 | 6 | 6 | 6 | 5 | 6 | 6 | 7 | 6 | 6 | 6 |
| ko00760 Nicotinate and nicotinamide metabolism | 13 | 16 | 14 | 13 | 17 | 16 | 15 | 17 | 14 | 15 | 17 | 15 | 16 | 16 |
| ko00770 Pantothenate and CoA biosynthesis | 14 | 16 | 16 | 13 | 17 | 14 | 13 | 15 | 14 | 13 | 15 | 14 | 13 | 15 |
| ko00780 Biotin metabolism | 2 | 6 | 6 | 2 | 5 | 1 | 1 | 1 | 1 | 1 | 1 | 5 | 1 | 1 |
| ko00785 Lipoic acid metabolism | 2 | 2 | 2 | 2 | 2 | 2 | 2 | 2 | 2 | 2 | 2 | 2 | 2 | 2 |
| ko00790 Folate biosynthesis | 9 | 9 | 7 | 7 | 9 | 8 | 10 | 10 | 8 | 8 | 9 | 9 | 9 | 9 |
| ko00670 One carbon pool by folate | 13 | 11 | 12 | 12 | 11 | 13 | 13 | 13 | 12 | 13 | 13 | 11 | 11 | 12 |
| ko00830 Retinol metabolism | - | 2 | 2 | 2 | 2 | 2 | 2 | 2 | 1 | 1 | 2 | 2 | 2 | 2 |
| ko00130 Ubiquinone and other terpenoid-quinone biosynthesis | 7 | 10 | 8 | 8 | 6 | 9 | 7 | 6 | 7 | 7 | 8 | 8 | 8 | 8 |
| ko00860 Porphyrin and chlorophyll metabolism | 32 | 36 | 51 | 53 | 37 | 16 | 35 | 34 | 34 | 33 | 35 | 32 | 33 | 35 |
| **1.9 Biosynthesis of Polyketides and Terpenoids** | **29** | **29** | **42** | **43** | **29** | **27** | **28** | **36** | **28** | **27** | **34** | **27** | **27** | **34** |
| ko01051 Biosynthesis of ansamycins | - | 1 | 2 | 1 | 1 | 1 | 1 | 2 | 1 | 1 | 2 | 1 | 1 | 2 |
| ko00253 Tetracycline biosynthesis | - | 4 | 4 | 4 | 4 | 4 | 4 | 4 | 4 | 4 | 4 | 4 | 4 | 4 |
| ko00523 Polyketide sugar unit biosynthesis | 4 | 1 | 4 | 4 | 4 | 4 | 4 | 4 | 4 | 4 | 4 | 4 | 4 | 4 |
| ko01053 Biosynthesis of siderophore group  nonribosomal peptides | - | 1 | 1 | 1 | - | - | 1 | 1 | 1 | 1 | 1 | 2 | 1 | 1 |

**Table S1.** *Cont.*

| **Relevant KEGG Pathways** | **KEGG ORTHOLOGS** | | | | | | | | | | | | | |
| --- | --- | --- | --- | --- | --- | --- | --- | --- | --- | --- | --- | --- | --- | --- |
|  | **azc** | **bja** | **bbt** | **bra** | **mlo** | **mes** | **ret** | **rec** | **rlg** | **rlt** | **rle** | **rhi** | **smd** | **sme** |
| **1.9 Biosynthesis of Polyketides and Terpenoids** | **29** | **29** | **42** | **43** | **29** | **27** | **28** | **36** | **28** | **27** | **34** | **27** | **27** | **34** |
| ko01055 Biosynthesis of vancomycin group antibiotics | - | 1 | 1 | 1 | 1 | 1 | 1 | 1 | 1 | 1 | 1 | 1 | 1 | 1 |
| ko00900 Terpenoid backbone biosynthesis | 10 | 10 | 12 | 12 | 10 | 10 | 11 | 12 | 10 | 10 | 10 | 10 | 10 | 10 |
| ko00903 Limonene and pinene degradation | 15 | 9 | 12 | 12 | 7 | 5 | 4 | 10 | 5 | 4 | 10 | 3 | 4 | 10 |
| ko00908 Zeatin biosynthesis | - | 1 | 1 | 1 | 1 | 1 | 1 | 1 | 1 | 1 | 1 | 1 | 1 | 1 |
| ko00906 Carotenoid biosynthesis | - | 1 | 5 | 7 | 1 | 1 | 1 | 1 | 1 | 1 | 1 | 1 | 1 | 1 |
| **1.10 Biosynthesis of Other Secondary Metabolites** | **8** | **21** | **32** | **35** | **31** | **20** | **27** | **34** | **26** | **26** | **37** | **26** | **23** | **28** |
| ko00940 Phenylpropanoid biosynthesis | - | 2 | 2 | 2 | 1 | 1 | 3 | 3 | 3 | 3 | 3 | 3 | 2 | 2 |
| ko00945 Stilbenoid, diarylheptanoid and gingerol biosynthesis | - | 1 | 1 | 1 | 1 | - | - | 1 | - | - | 1 | - | - | 1 |
| ko00941 Flavonoid biosynthesis | - | 1 | 2 | 2 | - | - | - | 1 | 1 | 1 | 2 | - | - | - |
| ko00901 Indole alkaloid biosynthesis | - | - | - | - | - | - | - | 1 | - | - | - | - | - | - |
| ko00950 Isoquinoline alkaloid biosynthesis | - | 2 | 4 | 4 | 5 | 1 | 3 | 4 | 3 | 3 | 5 | 3 | 2 | 3 |
| ko00960 Tropane, piperidine and pyridine alkaloid biosynthesis | - | 5 | 7 | 8 | 5 | 3 | 4 | 6 | 4 | 4 | 8 | 4 | 4 | 4 |
| ko00232 Caffeine metabolism | - | - | 1 | 1 | 1 | - | 1 | 1 | - | - | - | - | - | 1 |
| ko00965 Betalain biosynthesis | - | - | - | - | 1 | - | - | 1 | - | - | - | 1 | - | - |
| ko00311 Penicillin and cephalosporin biosynthesis | - | 1 | 1 | 2 | 2 | 1 | 2 | 1 | 2 | 2 | 2 | 1 | 1 | 1 |
| ko00312 beta-Lactam resistance | - | 1 | 1 | 1 |  | 1 | 1 | 1 | 1 | 1 | 1 | 1 | 1 | 1 |
| ko00521 Streptomycin biosynthesis | 6 | 4 | 8 | 9 | 8 | 8 | 9 | 9 | 8 | 8 | 8 | 8 | 9 | 9 |
| ko00901 Indole alkaloid biosynthesis | - | - | - | - | 1 | - | - | - | - | - | - | 1 | - | - |
| ko00524 Butirosin and neomycin biosynthesis | - | 1 | 1 | 1 | 1 | 1 | 1 | 1 | 1 | 1 | 1 | 1 | 1 | 1 |
| ko00401 Novobiocin biosynthesis | 2 | 3 | 4 | 4 | 5 | 4 | 3 | 4 | 3 | 3 | 6 | 3 | 3 | 5 |
| **1.11 Xenobiotics Biodegradation and Metabolism** | **85** | **134** | **166** | **163** | **97** | **72** | **58** | **129** | **60** | **59** | **138** | **55** | **53** | **101** |
| ko00930 Caprolactam degradation | 2 | 5 | 5 | 4 | 3 | 3 | 4 | 5 | 4 | 4 | 5 | 3 | 3 | 4 |

**Table S1.** *Cont.*

| **Relevant KEGG Pathways** | **KEGG ORTHOLOGS** | | | | | | | | | | | | | |
| --- | --- | --- | --- | --- | --- | --- | --- | --- | --- | --- | --- | --- | --- | --- |
|  | **azc** | **bja** | **bbt** | **bra** | **mlo** | **mes** | **ret** | **rec** | **rlg** | **rlt** | **rle** | **rhi** | **smd** | **sme** |
| **1.11 Xenobiotics Biodegradation and Metabolism** | **85** | **134** | **166** | **163** | **97** | **72** | **58** | **129** | **60** | **59** | **138** | **55** | **53** | **101** |
| ko00621 Biphenyl degradation | 2 | - | 1 | 1 | - | - | 1 | 2 | 1 | 1 | 2 | - | - | - |
| ko00622 Toluene and xylene degradation | - | 6 | 10 | 8 | 3 | 2 | 1 | 5 | 2 | 2 | 7 | 1 | - | 3 |
| ko00361 gamma-Hexachlorocyclohexane degradation | 4 | 10 | 10 | 11 | 7 | 2 | 3 | 11 | 3 | 3 | 12 | 3 | 2 | 6 |
| ko00641 3-Chloroacrylic acid degradation | - | 5 | 5 | 5 | 5 | 4 | 3 | 5 | 2 | 2 | 5 | 3 | 3 | 4 |
| ko00351 1,1,1-Trichloro-2,2-bis(4-chlorophenyl)ethane (DDT) degradation | - | 1 | 3 | 3 | - | 1 | - | 2 | - | - | - | - | - | 1 |
| ko00623 2,4-Dichlorobenzoate degradation | 7 | 9 | 9 | 8 | 3 | 4 | 3 | 5 | 3 | 3 | 4 | 3 | 3 | 3 |
| ko00631 1,2-Dichloroethane degradation | - | 5 | 4 | 4 | 3 | 2 | 2 | 4 | 2 | 2 | 3 | 2 | 2 | 3 |
| ko00625 Tetrachloroethene degradation | 7 | 4 | 4 | 4 | 4 |  | 3 | 4 | 3 | 3 | 4 | 3 | 3 | 4 |
| ko00643 Styrene degradation | 4 | 9 | 8 | 8 | 6 | 5 | 4 | 5 | 4 | 4 | 7 | 5 | 4 | 5 |
| ko00627 1,4-Dichlorobenzene degradation | 14 | 6 | 7 | 8 | 4 | 2 | 2 | 5 | 3 | 3 | 8 | 3 | 2 | 4 |
| ko00626 Naphthalene and anthracene degradation | 4 | 6 | 9 | 9 | 5 | 3 | - | 8 | - | - | 8 | - | - | 6 |
| ko00642 Ethylbenzene degradation | 2 | 1 | 1 | 1 | 2 | 1 | - | 2 | - | - | 2 | - | - | 2 |
| ko00628 Fluorene degradation | - | 4 | 7 | 6 | 3 | 2 | 1 | 4 | 1 | 1 | 5 | 1 | 1 | 3 |
| ko00629 Carbazole degradation | - | 1 | 2 | 3 | - | - | - | 1 | 1 | 1 | 2 | 1 | - | - |
| ko00632 Benzoate degradation via CoA ligation | - | 17 | 18 | 18 | 12 | 10 | 11 | 14 | 11 | 11 | 14 | 10 | 11 | 14 |
| ko00362 Benzoate degradation via hydroxylation | 20 | 17 | 23 | 23 | 12 | 12 | 10 | 17 | 10 | 9 | 20 | 6 | 8 | 12 |
| ko00791 Atrazine degradation | 3 | 5 | 7 | 6 | 3 | 3 | 3 | 6 | 4 | 4 | 6 | 3 | 3 | 4 |
| ko00363 Bisphenol A degradation | - | 2 | 4 | 4 | 2 | - | - | 3 | - | - | 3 | - | - | 3 |
| ko00624 1- and 2-Methylnaphthalene degradation | 4 | 9 | 11 | 12 | 8 | 6 | 2 | 10 | 1 | 1 | 9 | 2 | 2 | 10 |
| ko00633 Trinitrotoluene degradation | 6 | 5 | 7 | 7 | 5 | 3 | 2 | 2 | 1 | 1 | 3 | 2 | 3 | 2 |
| ko00281 Geraniol degradation | 3 | 4 | 6 | 6 | 5 | 5 | 2 | 6 | 2 | 2 | 5 | 2 | 2 | 6 |
| ko00364 Fluorobenzoate degradation | 3 | 3 | 5 | 4 | 2 | 2 | 1 | 3 | 2 | 2 | 4 | 2 | 1 | 2 |

**Table S1.** *Cont.*

| **Relevant KEGG Pathways** | **KEGG ORTHOLOGS** | | | | | | | | | | | | | |
| --- | --- | --- | --- | --- | --- | --- | --- | --- | --- | --- | --- | --- | --- | --- |
|  | **azc** | **bja** | **bbt** | **bra** | **mlo** | **mes** | **ret** | **rec** | **rlg** | **rlt** | **rle** | **rhi** | **smd** | **sme** |
| **2. Genetic Information Processing** | **247** | **249** | **241** | **242** | **240** | **231** | **239** | **240** | **244** | **242** | **241** | **234** | **242** | **245** |
| **2.1 Transcription** | **4** | **4** | **4** | **4** | **4** | **4** | **4** | **4** | **4** | **4** | **4** | **4** | **4** | **4** |
| ko03020 RNA polymerase | 4 | 4 | 4 | 4 | 4 | 4 | 4 | 4 | 4 | 4 | 4 | 3 | 4 | 4 |
| **2.2 Translation** | **134** | **131** | **128** | **129** | **130** | **123** | **128** | **128** | **129** | **129** | **127** | **124** | **129** | **132** |
| ko03010 Ribosome | 57 | 54 | 53 | 54 | 57 | 54 | 54 | 54 | 54 | 54 | 51 | 52 | 54 | 57 |
| ko00970 Aminoacyl-tRNA biosynthesis | 47 | 46 | 47 | 47 | 46 | 44 | 46 | 46 | 47 | 47 | 47 | 45 | 47 | 47 |
| ko03060 Protein export | 16 | 17 | 16 | 16 | 14 | 15 | 15 | 15 | 15 | 15 | 15 | 14 | 15 | 15 |
| ko03018 RNA degradation | 14 | 14 | 12 | 12 | 13 | 10 | 13 | 13 | 13 | 13 | 14 | 13 | 13 | 13 |
| **2.3 Folding, Sorting and Degradation** | **40** | **40** | **38** | **38** | **36** | **34** | **36** | **37** | **38** | **38** | **37** | **36** | **38** | **38** |
| ko03060 Protein Export | 16 | 17 | 17 | 17 | 14 | 15 | 15 | 15 | 16 | 16 | 15 | 14 | 16 | 16 |
| ko04122 Sulfur relay system | 10 | 9 | 9 | 9 | 9 | 9 | 8 | 8 | 8 | 8 | 8 | 8 | 8 | 8 |
| ko03018 RNA degredation | 14 | 14 | 12 | 12 | 13 | 10 | 13 | 14 | 14 | 14 | 14 | 14 | 14 | 14 |
| **2.4 Replication and Repair** | **69** | **74** | **71** | **71** | **70** | **70** | **71** | **71** | **73** | **71** | **73** | **70** | **71** | **71** |
| ko03030 DNA replication | 14 | 14 | 14 | 14 | 14 | 14 | 14 | 14 | 14 | 14 | 14 | 14 | 14 | 14 |
| ko03410 Base excision repair | 10 | 13 | 12 | 12 | 11 | 11 | 12 | 12 | 13 | 12 | 13 | 11 | 12 | 12 |
| ko03420 Nucleotide excision repair | 8 | 8 | 8 | 8 | 8 | 8 | 8 | 8 | 8 | 8 | 8 | 8 | 8 | 8 |
| ko03430 Mismatch repair | 16 | 17 | 16 | 16 | 16 | 16 | 16 | 16 | 17 | 16 | 17 | 16 | 16 | 16 |
| ko03440 Homologous recombination | 19 | 20 | 19 | 19 | 19 | 19 | 19 | 19 | 19 | 19 | 19 | 19 | 19 | 19 |
| ko03450 Non-homologous end-joining | 2 | 2 | 2 | 2 | 2 | 2 | 2 | 2 | 2 | 2 | 2 | 2 | 2 | 2 |
| **3. Environmental Information Processing** | **176** | **202** | **180** | **171** | **222** | **174** | **209** | **218** | **186** | **179** | **215** | **214** | **194** | **219** |
| **3.1 Membrane Transport** | **119** | **141** | **125** | **114** | **172** | **135** | **159** | **163** | **138** | **131** | **162** | **165** | **148** | **170** |
| ko02010 ABC transporters | 88 | 91 | 85 | 84 | 119 | 97 | 117 | 127 | 112 | 109 | 132 | 120 | 120 | 141 |
| ko02060 Phosphotransferase system | 3 | 4 | 3 | 4 | 7 | 3 | 4 | 3 | 4 | 3 | 4 | 4 | 3 | 3 |
| ko03070 Bacterial secretion system | 28 | 46 | 37 | 26 | 46 | 35 | 38 | 33 | 22 | 19 | 26 | 41 | 25 | 26 |

**Table S1.** *Cont.*

| **Relevant KEGG Pathways** | **KEGG ORTHOLOGS** | | | | | | | | | | | | | |
| --- | --- | --- | --- | --- | --- | --- | --- | --- | --- | --- | --- | --- | --- | --- |
|  | **azc** | **bja** | **bbt** | **bra** | **mlo** | **mes** | **ret** | **rec** | **rlg** | **rlt** | **rle** | **rhi** | **smd** | **sme** |
| **3.2 Signal Transduction** | **57** | **61** | **55** | **57** | **50** | **39** | **50** | **55** | **48** | **48** | **53** | **49** | **46** | **49** |
| ko02020 Two-component system | 57 | 61 | 55 | 57 | 50 | 39 | 50 | 55 | 48 | 48 | 53 | 49 | 46 | 49 |
| **4. Cellular Processes** | **38** | **41** | **43** | **43** | **34** | **38** | **40** | **41** | **40** | **40** | **41** | **40** | **40** | **40** |
| **4.2 Cell Motility** | **38** | **41** | **43** | **43** | **34** | **38** | **40** | **41** | **40** | **40** | **41** | **40** | **40** | **40** |
| ko02030 Bacterial chemotaxis | 13 | 13 | 15 | 15 | 9 | 13 | 15 | 16 | 15 | 15 | 16 | 15 | 15 | 15 |
| ko02040 Flagellar assembly | 25 | 28 | 28 | 28 | 25 | 25 | 25 | 25 | 25 | 25 | 25 | 25 | 25 | 25 |

**Table S2.** Nitrogen metabolism KEGG orthologs.

| **#K0** | **Cluster** | **Coding gene** | **product** | **azc** | **bja** | **bbt** | **bra** | **mlo** | **mes** | **ret** | **rec** | **rlg** | **rlt** | **rle** | **rhi** | **smd** | **sme** |
| --- | --- | --- | --- | --- | --- | --- | --- | --- | --- | --- | --- | --- | --- | --- | --- | --- | --- |
| K00260 | [1951](http://ccg.murdoch.edu.au/organism/rhizobium/RhizobiumSummaries/queryClusters/msf/Cluster1951.msf) | *gdhB* | glutamate dehydrogenase |  |  |  |  |  |  |  |  |  |  |  |  |  |  |
| K00261 | [4283](http://ccg.murdoch.edu.au/organism/rhizobium/RhizobiumSummaries/queryClusters/msf/Cluster4283.msf) |  | glutamate dehydrogenase (NAD(P)+) |  |  |  |  |  |  |  |  |  |  |  |  |  |  |
| K00262 | [21243](http://ccg.murdoch.edu.au/organism/rhizobium/RhizobiumSummaries/queryClusters/msf/Cluster21243.msf) | *gdhA* | glutamate dehydrogenase |  |  |  |  |  |  |  |  |  |  |  |  |  |  |
| K00265 | [1033](http://ccg.murdoch.edu.au/organism/rhizobium/RhizobiumSummaries/queryClusters/msf/Cluster1033.msf) | *gltB* | glutamate synthase (NADPH/NADH) large chain |  |  |  |  |  |  |  |  |  |  |  |  |  |  |
| K00266 | [1034](http://ccg.murdoch.edu.au/organism/rhizobium/RhizobiumSummaries/queryClusters/msf/Cluster1034.msf) | *gltD* | glutamate synthase (NADPH/NADH) small chain |  |  |  |  |  |  |  |  |  |  |  |  |  |  |
| K00285 | [110](http://ccg.murdoch.edu.au/organism/rhizobium/RhizobiumSummaries/queryClusters/msf/Cluster110.msf) | *dadA* | D-amino-acid dehydrogenase |  |  |  |  |  |  |  |  |  |  |  |  |  |  |
| K00362 | [91](http://ccg.murdoch.edu.au/organism/rhizobium/RhizobiumSummaries/queryClusters/msf/Cluster91.msf) | *nirB* | nitrite reductase (NAD(P)H) large subunit |  |  |  |  |  |  |  |  |  |  |  |  |  |  |
| K00363 | [2427](http://ccg.murdoch.edu.au/organism/rhizobium/RhizobiumSummaries/queryClusters/msf/Cluster2427.msf) | *nirD* | nitrite reductase (NAD(P)H) small subunit [EC:1.7.1.4] |  |  |  |  |  |  |  |  |  |  |  |  |  |  |
| K00366 | [4938](http://ccg.murdoch.edu.au/organism/rhizobium/RhizobiumSummaries/queryClusters/msf/Cluster4938.msf) | *nirA* | ferredoxin-nitrite reductase [EC:1.7.7.1] |  |  |  |  |  |  |  |  |  |  |  |  |  |  |
| K00368 | [2243](http://ccg.murdoch.edu.au/organism/rhizobium/RhizobiumSummaries/queryClusters/msf/Cluster2243.msf) |  | nitrite reductase (NO-forming) [EC:1.7.2.1] |  |  |  |  |  |  |  |  |  |  |  |  |  |  |
| K00369 | [19160](http://ccg.murdoch.edu.au/organism/rhizobium/RhizobiumSummaries/queryClusters/seqs/Cluster19160.fa) |  | nitrate reductase [EC:1.7.99.4] |  |  |  |  |  |  |  |  |  |  |  |  |  |  |

**Table S2.** *Cont.*

| **#K0** | **Cluster** | **Coding gene** | **product** | **azc** | **bja** | **bbt** | **bra** | **mlo** | **mes** | **ret** | **rec** | **rlg** | **rlt** | **rle** | **rhi** | **smd** | **sme** |
| --- | --- | --- | --- | --- | --- | --- | --- | --- | --- | --- | --- | --- | --- | --- | --- | --- | --- |
| K00371 | [10313](http://ccg.murdoch.edu.au/organism/rhizobium/RhizobiumSummaries/queryClusters/seqs/Cluster10313.fa) | *narB/narY* | nitrate reductase 1, beta subunit [EC:1.7.99.4] |  |  |  |  |  |  |  |  |  |  |  |  |  |  |
| K00372 | [1063](http://ccg.murdoch.edu.au/organism/rhizobium/RhizobiumSummaries/queryClusters/msf/Cluster1063.msf) | *nasA* | nitrate reductase catalytic subunit [EC:1.7.99.4] |  |  |  |  |  |  |  |  |  |  |  |  |  |  |
| K00373 | [10314](http://ccg.murdoch.edu.au/organism/rhizobium/RhizobiumSummaries/queryClusters/seqs/Cluster10314.fa) | *narJ* | nitrate reductase 1, delta subunit [EC:1.7.99.4] |  |  |  |  |  |  |  |  |  |  |  |  |  |  |
| K00374 | [10315](http://ccg.murdoch.edu.au/organism/rhizobium/RhizobiumSummaries/queryClusters/seqs/Cluster10315.fa) | *narI* | nitrate reductase 1, gamma subunit [EC:1.7.99.4] |  |  |  |  |  |  |  |  |  |  |  |  |  |  |
| K00376 | [6625](http://ccg.murdoch.edu.au/organism/rhizobium/RhizobiumSummaries/queryClusters/msf/Cluster6625.msf) | *nosR* | nitrous-oxide reductase [EC:1.7.99.6] |  |  |  |  |  |  |  |  |  |  |  |  |  |  |
| K00459 | [706](http://ccg.murdoch.edu.au/organism/rhizobium/RhizobiumSummaries/queryClusters/msf/Cluster706.msf) |  | nitronate monooxygenase [EC:1.13.12.16] |  |  |  |  |  |  |  |  |  |  |  |  |  |  |
| K00605 | [891](http://ccg.murdoch.edu.au/organism/rhizobium/RhizobiumSummaries/queryClusters/msf/Cluster891.msf) | *gcvT* | aminomethyltransferase [EC:2.1.2.10] |  |  |  |  |  |  |  |  |  |  |  |  |  |  |
| K00926 | [4431](http://ccg.murdoch.edu.au/organism/rhizobium/RhizobiumSummaries/queryClusters/msf/Cluster4431.msf) | *arcC* | carbamate kinase [EC:2.7.2.2] |  |  |  |  |  |  |  |  |  |  |  |  |  |  |
| K01424 | [5986](http://ccg.murdoch.edu.au/organism/rhizobium/RhizobiumSummaries/queryClusters/msf/Cluster5986.msf) | *ansA* | L-asparaginase [EC:3.5.1.1] |  |  |  |  |  |  |  |  |  |  |  |  |  |  |
| K01425 | [1394](http://ccg.murdoch.edu.au/organism/rhizobium/RhizobiumSummaries/queryClusters/msf/Cluster1394.msf) | *glsA* | glutaminase [EC:3.5.1.2] |  |  |  |  |  |  |  |  |  |  |  |  |  |  |
| K01667 | [19856](http://ccg.murdoch.edu.au/organism/rhizobium/RhizobiumSummaries/queryClusters/seqs/Cluster19856.fa) | *tnaA* | tryptophanase [EC:4.1.99.1] |  |  |  |  |  |  |  |  |  |  |  |  |  |  |
| K01455 | [4215](http://ccg.murdoch.edu.au/organism/rhizobium/RhizobiumSummaries/queryClusters/msf/Cluster4215.msf) |  | formamidase [EC:3.5.1.49] |  |  |  |  |  |  |  |  |  |  |  |  |  |  |
| K01501 | [7648](http://ccg.murdoch.edu.au/organism/rhizobium/RhizobiumSummaries/queryClusters/msf/Cluster7648.msf) |  | nitrilase [EC:3.5.5.1] |  |  |  |  |  |  |  |  |  |  |  |  |  |  |
| K01673 | [622](http://ccg.murdoch.edu.au/organism/rhizobium/RhizobiumSummaries/queryClusters/msf/Cluster622.msf) | *cynT* | carbonic anhydrase [EC:4.2.1.1] |  |  |  |  |  |  |  |  |  |  |  |  |  |  |
| K01674 | [3373](http://ccg.murdoch.edu.au/organism/rhizobium/RhizobiumSummaries/queryClusters/msf/Cluster3373.msf) | *cah* | carbonic anhydrase [EC:4.2.1.1] |  |  |  |  |  |  |  |  |  |  |  |  |  |  |
| K01725 | [4944](http://ccg.murdoch.edu.au/organism/rhizobium/RhizobiumSummaries/queryClusters/msf/Cluster4944.msf) | *cynS* | cyanate lyase [EC:4.2.1.104] |  |  |  |  |  |  |  |  |  |  |  |  |  |  |
| K01744 | [265](http://ccg.murdoch.edu.au/organism/rhizobium/RhizobiumSummaries/queryClusters/msf/Cluster265.msf) | *aspA* | aspartate ammonia-lyase [EC:4.3.1.1] |  |  |  |  |  |  |  |  |  |  |  |  |  |  |
| K01745 | [200](http://ccg.murdoch.edu.au/organism/rhizobium/RhizobiumSummaries/queryClusters/msf/Cluster200.msf) | *hutH* | histidine ammonia-lyase [EC:4.3.1.3] |  |  |  |  |  |  |  |  |  |  |  |  |  |  |
| K01758 | [16459](http://ccg.murdoch.edu.au/organism/rhizobium/RhizobiumSummaries/queryClusters/seqs/Cluster16459.fa) |  | cystathionine gamma-lyase [EC:4.4.1.1] |  |  |  |  |  |  |  |  |  |  |  |  |  |  |
| K01760 | [34](http://ccg.murdoch.edu.au/organism/rhizobium/RhizobiumSummaries/queryClusters/msf/Cluster34.msf) | *metC* | cystathionine beta-lyase [EC:4.4.1.8] |  |  |  |  |  |  |  |  |  |  |  |  |  |  |
| K01915 | [270](http://ccg.murdoch.edu.au/organism/rhizobium/RhizobiumSummaries/queryClusters/msf/Cluster270.msf) | *glnA* | glutamine synthetase [EC:6.3.1.2] |  |  |  |  |  |  |  |  |  |  |  |  |  |  |
| K01916 | [3991](http://ccg.murdoch.edu.au/organism/rhizobium/RhizobiumSummaries/queryClusters/msf/Cluster3991.msf) | *nadE* | NAD+ synthase [EC:6.3.1.5] |  |  |  |  |  |  |  |  |  |  |  |  |  |  |

**Table S2.** *Cont.*

| **#K0** | **Cluster** | **Coding gene** | **product** | **azc** | **bja** | **bbt** | **bra** | **mlo** | **mes** | **ret** | **rec** | **rlg** | **rlt** | **rle** | **rhi** | **smd** | **sme** |
| --- | --- | --- | --- | --- | --- | --- | --- | --- | --- | --- | --- | --- | --- | --- | --- | --- | --- |
| K01953 | [666](http://ccg.murdoch.edu.au/organism/rhizobium/RhizobiumSummaries/queryClusters/msf/Cluster666.msf) | *asnB* | asparagine synthase (glutamine-hydrolysing) [EC:6.3.5.4] |  |  |  |  |  |  |  |  |  |  |  |  |  |  |
| K02164 | [3392](http://ccg.murdoch.edu.au/organism/rhizobium/RhizobiumSummaries/queryClusters/msf/Cluster3392.msf) | *norE* | nitric-oxide reductase NorE protein [EC:1.7.99.7] |  |  |  |  |  |  |  |  |  |  |  |  |  |  |
| K02305 | [3393](http://ccg.murdoch.edu.au/organism/rhizobium/RhizobiumSummaries/queryClusters/msf/Cluster3393.msf) | *norC* | nitric-oxide reductase, cytochrome c-containing subunit II [EC:1.7.99.7] |  |  |  |  |  |  |  |  |  |  |  |  |  |  |
| K02448 | [3394](http://ccg.murdoch.edu.au/organism/rhizobium/RhizobiumSummaries/queryClusters/msf/Cluster3394.msf) | *norD* | nitric-oxide reductase NorD protein [EC:1.7.99.7] |  |  |  |  |  |  |  |  |  |  |  |  |  |  |
| K02567 | [3389](http://ccg.murdoch.edu.au/organism/rhizobium/RhizobiumSummaries/queryClusters/msf/Cluster3389.msf) | *napA* | periplasmic nitrate reductase NapA [EC:1.7.99.4] |  |  |  |  |  |  |  |  |  |  |  |  |  |  |
| K02586 | [190](http://ccg.murdoch.edu.au/organism/rhizobium/RhizobiumSummaries/queryClusters/msf/Cluster190.msf) | *nifD* | nitrogenase molybdenum-iron protein alpha chain [EC:1.18.6.1] |  |  |  |  |  |  |  |  |  |  |  |  |  |  |
| K02588 | [307](http://ccg.murdoch.edu.au/organism/rhizobium/RhizobiumSummaries/queryClusters/msf/Cluster307.msf) | *nifH* | nitrogenase iron protein NifH [EC:1.18.6.1] |  |  |  |  |  |  |  |  |  |  |  |  |  |  |
| K02591 | [213](http://ccg.murdoch.edu.au/organism/rhizobium/RhizobiumSummaries/queryClusters/msf/Cluster213msf) | *nifK* | nitrogenase molybdenum-iron protein beta chain [EC:1.18.6.1] |  |  |  |  |  |  |  |  |  |  |  |  |  |  |
| K04561 | [3394](http://ccg.murdoch.edu.au/organism/rhizobium/RhizobiumSummaries/queryClusters/msf/Cluster3394msf) | *norB* | nitric-oxide reductase, cytochrome b-containing subunit I [EC:1.7.99.7] |  |  |  |  |  |  |  |  |  |  |  |  |  |  |
| K04748 | [2653](http://ccg.murdoch.edu.au/organism/rhizobium/RhizobiumSummaries/queryClusters/msf/Cluster3394msf) | *norQ* | nitric-oxide reductase NorQ protein [EC:1.7.99.7] |  |  |  |  |  |  |  |  |  |  |  |  |  |  |
| K04835 | [16420](http://ccg.murdoch.edu.au/organism/rhizobium/RhizobiumSummaries/queryClusters/seqs/Cluster16420.fa) |  | methylaspartate ammonia-lyase [EC:4.3.1.2] |  |  |  |  |  |  |  |  |  |  |  |  |  |  |
| K07256 | [21185](http://ccg.murdoch.edu.au/organism/rhizobium/RhizobiumSummaries/queryClusters/seqs/Cluster21185.fa) | *tauY* | taurine dehydrogenase large subunit [EC:1.4.2.-] |  |  |  |  |  |  |  |  |  |  |  |  |  |  |
|  | Nitrogenase complex genes | | | | | | | | | | | | | | | | |

* The gray color indicates absence of the gene.

**Table S3.** Nod protein orthologs.

| **Coding gene** | **Cluster** | **azc** | **bja** | **bbt** | **bra** | **mlo** | **mes** | **ret** | **rec** | **rlg** | **rlt** | **rle** | **rhi** | **smd** | **sme** |
| --- | --- | --- | --- | --- | --- | --- | --- | --- | --- | --- | --- | --- | --- | --- | --- |
| *nodA* | [2102](http://ccg.murdoch.edu.au/organism/rhizobium/RhizobiumSummaries/queryClusters/msf/Cluster2102.msf) |  |  |  |  |  |  |  |  |  |  |  |  |  |  |
| *nodB* | [1929](http://ccg.murdoch.edu.au/organism/rhizobium/RhizobiumSummaries/queryClusters/msf/Cluster1929.msf) |  |  |  |  |  |  |  |  |  |  |  |  |  |  |
| *nodC* | [2101](http://ccg.murdoch.edu.au/organism/rhizobium/RhizobiumSummaries/queryClusters/msf/Cluster2101.msf) |  |  |  |  |  |  |  |  |  |  |  |  |  |  |
| *nodD* | [104](http://ccg.murdoch.edu.au/organism/rhizobium/RhizobiumSummaries/queryClusters/msf/Cluster104.msf) |  |  |  |  |  |  |  |  |  |  |  |  |  |  |
| *nodE* | [66](http://ccg.murdoch.edu.au/organism/rhizobium/RhizobiumSummaries/queryClusters/msf/Cluster66.msf) |  |  |  |  |  |  |  |  |  |  |  |  |  |  |
| *nodF* | [3950](http://ccg.murdoch.edu.au/organism/rhizobium/RhizobiumSummaries/queryClusters/msf/Cluster3950.msf) |  |  |  |  |  |  |  |  |  |  |  |  |  |  |
| *nodG* | [2](http://ccg.murdoch.edu.au/organism/rhizobium/RhizobiumSummaries/queryClusters/msf/Cluster2.msf) |  |  |  |  |  |  |  |  |  |  |  |  |  |  |
| *nodH* | [9911](http://ccg.murdoch.edu.au/organism/rhizobium/RhizobiumSummaries/queryClusters/msf/Cluster9911.msf) |  |  |  |  |  |  |  |  |  |  |  |  |  |  |
| *nodI* | [109](http://ccg.murdoch.edu.au/organism/rhizobium/RhizobiumSummaries/queryClusters/msf/Cluster109.msf) |  |  |  |  |  |  |  |  |  |  |  |  |  |  |
| *nodJ* | [2100](http://ccg.murdoch.edu.au/organism/rhizobium/RhizobiumSummaries/queryClusters/msf/Cluster2100.msf) |  |  |  |  |  |  |  |  |  |  |  |  |  |  |
| *nodL* | [1854](http://ccg.murdoch.edu.au/organism/rhizobium/RhizobiumSummaries/queryClusters/msf/Cluster1854.msf) |  |  |  |  |  |  |  |  |  |  |  |  |  |  |
| *nodM* | [383](http://ccg.murdoch.edu.au/organism/rhizobium/RhizobiumSummaries/queryClusters/msf/Cluster383.msf) |  |  |  |  |  |  |  |  |  |  |  |  |  |  |
| *nodN* | [526](http://ccg.murdoch.edu.au/organism/rhizobium/RhizobiumSummaries/queryClusters/msf/Cluster526.msf) |  |  |  |  |  |  |  |  |  |  |  |  |  |  |
| *nodO* | [19055](http://ccg.murdoch.edu.au/organism/rhizobium/RhizobiumSummaries/queryClusters/seqs/Cluster19055.fa) |  |  |  |  |  |  |  |  |  |  |  |  |  |  |
| *nodP* | [404](http://ccg.murdoch.edu.au/organism/rhizobium/RhizobiumSummaries/queryClusters/msf/Cluster404.msf) |  |  |  |  |  |  |  |  |  |  |  |  |  |  |
| *nodQ* | [374](http://ccg.murdoch.edu.au/organism/rhizobium/RhizobiumSummaries/queryClusters/msf/Cluster374.msf) |  |  |  |  |  |  |  |  |  |  |  |  |  |  |
| *nodR* | [19236](http://ccg.murdoch.edu.au/organism/rhizobium/RhizobiumSummaries/queryClusters/seqs/Cluster19236.fa) |  |  |  |  |  |  |  |  |  |  |  |  |  |  |
| *nodS* | [3695](http://ccg.murdoch.edu.au/organism/rhizobium/RhizobiumSummaries/queryClusters/msf/Cluster3695.msf) |  |  |  |  |  |  |  |  |  |  |  |  |  |  |
| *nodT* | [418](http://ccg.murdoch.edu.au/organism/rhizobium/RhizobiumSummaries/queryClusters/msf/Cluster418.msf) |  |  |  |  |  |  |  |  |  |  |  |  |  |  |
| *nodU* | [5971](http://ccg.murdoch.edu.au/organism/rhizobium/RhizobiumSummaries/queryClusters/msf/Cluster5971msf) |  |  |  |  |  |  |  |  |  |  |  |  |  |  |
| *nodV* | [82](http://ccg.murdoch.edu.au/organism/rhizobium/RhizobiumSummaries/queryClusters/msf/Cluster82.msf) |  |  |  |  |  |  |  |  |  |  |  |  |  |  |
| *nodW* | [63](http://ccg.murdoch.edu.au/organism/rhizobium/RhizobiumSummaries/queryClusters/msf/Cluster63.msf) |  |  |  |  |  |  |  |  |  |  |  |  |  |  |
| *nodX* | [3712](http://ccg.murdoch.edu.au/organism/rhizobium/RhizobiumSummaries/queryClusters/msf/Cluster3712.msf) |  |  |  |  |  |  |  |  |  |  |  |  |  |  |
| *nodY* | [12393](http://ccg.murdoch.edu.au/organism/rhizobium/RhizobiumSummaries/queryClusters/seqs/Cluster12393.fa) |  |  |  |  |  |  |  |  |  |  |  |  |  |  |
| *nodZ* | [4207](http://ccg.murdoch.edu.au/organism/rhizobium/RhizobiumSummaries/queryClusters/msf/Cluster4207.msf) |  |  |  |  |  |  |  |  |  |  |  |  |  |  |

* The gray color indicates absence of the gene.

**Table S4.** Noe protein orthologs.

| **Coding gene** | **Cluster** | **azc** | **bja** | **bbt** | **bra** | **mlo** | **mes** | **ret** | **rec** | **rlg** | **rlt** | **rle** | **rhi** | **smd** | **sme** |
| --- | --- | --- | --- | --- | --- | --- | --- | --- | --- | --- | --- | --- | --- | --- | --- |
| *noeA* | [5425](http://ccg.murdoch.edu.au/organism/rhizobium/RhizobiumSummaries/queryClusters/msf/Cluster5425.msf) |  |  |  |  |  |  |  |  |  |  |  |  |  |  |
| *noeB* | [9915](http://ccg.murdoch.edu.au/organism/rhizobium/RhizobiumSummaries/queryClusters/msf/Cluster9915.msf) |  |  |  |  |  |  |  |  |  |  |  |  |  |  |
| *noeC* | [2980](http://ccg.murdoch.edu.au/organism/rhizobium/RhizobiumSummaries/queryClusters/msf/Cluster2980.msf) |  |  |  |  |  |  |  |  |  |  |  |  |  |  |
| *noeD* | [12248](http://ccg.murdoch.edu.au/organism/rhizobium/RhizobiumSummaries/queryClusters/msf/Cluster12248.msf) |  |  |  |  |  |  |  |  |  |  |  |  |  |  |
| *noeE* | [6845](http://ccg.murdoch.edu.au/organism/rhizobium/RhizobiumSummaries/queryClusters/msf/Cluster6845.msf) |  |  |  |  |  |  |  |  |  |  |  |  |  |  |
| *noeI* | [5361](http://ccg.murdoch.edu.au/organism/rhizobium/RhizobiumSummaries/queryClusters/msf/Cluster5361.msf) |  |  |  |  |  |  |  |  |  |  |  |  |  |  |
| *noeJ* | [400](http://ccg.murdoch.edu.au/organism/rhizobium/RhizobiumSummaries/queryClusters/msf/Cluster400.msf) |  |  |  |  |  |  |  |  |  |  |  |  |  |  |
| *noeK* | [400](http://ccg.murdoch.edu.au/organism/rhizobium/RhizobiumSummaries/queryClusters/msf/Cluster400.msf) |  |  |  |  |  |  |  |  |  |  |  |  |  |  |
| *noeL* | [428](http://ccg.murdoch.edu.au/organism/rhizobium/RhizobiumSummaries/queryClusters/msf/Cluster428.msf) |  |  |  |  |  |  |  |  |  |  |  |  |  |  |

* The gray color indicates absence of the gene.

**Table S5.** Nol protein orthologs.

| **Coding gene** | **Cluster** | **azc** | **bja** | **bbt** | **bra** | **mlo** | **mes** | **ret** | **rec** | **rlg** | **rlt** | **rle** | **rhi** | **smd** | **sme** |
| --- | --- | --- | --- | --- | --- | --- | --- | --- | --- | --- | --- | --- | --- | --- | --- |
| *nolA* | [12390](http://ccg.murdoch.edu.au/organism/rhizobium/RhizobiumSummaries/queryClusters/msf/Cluster12390.msf) |  |  |  |  |  |  |  |  |  |  |  |  |  |  |
| *nolB* | [12304](http://ccg.murdoch.edu.au/organism/rhizobium/RhizobiumSummaries/queryClusters/msf/Cluster12304.msf) |  |  |  |  |  |  |  |  |  |  |  |  |  |  |
| *nolE* | [9252](http://ccg.murdoch.edu.au/organism/rhizobium/RhizobiumSummaries/queryClusters/msf/Cluster9252.msf) |  |  |  |  |  |  |  |  |  |  |  |  |  |  |
| *nolF* | [9909](http://ccg.murdoch.edu.au/organism/rhizobium/RhizobiumSummaries/queryClusters/msf/Cluster9909.msf) |  |  |  |  |  |  |  |  |  |  |  |  |  |  |
| *nolG* | [23](http://ccg.murdoch.edu.au/organism/rhizobium/RhizobiumSummaries/queryClusters/msf/Cluster23.msf) |  |  |  |  |  |  |  |  |  |  |  |  |  |  |
| *nolK* | [745](http://ccg.murdoch.edu.au/organism/rhizobium/RhizobiumSummaries/queryClusters/msf/Cluster745.msf) |  |  |  |  |  |  |  |  |  |  |  |  |  |  |
| *nolL* | [7064](http://ccg.murdoch.edu.au/organism/rhizobium/RhizobiumSummaries/queryClusters/msf/Cluster7064.msf) |  |  |  |  |  |  |  |  |  |  |  |  |  |  |
| *nolM* | [12394](http://ccg.murdoch.edu.au/organism/rhizobium/RhizobiumSummaries/queryClusters/seqs/Cluster12394.fa) |  |  |  |  |  |  |  |  |  |  |  |  |  |  |
| *nolN* | [3832](http://ccg.murdoch.edu.au/organism/rhizobium/RhizobiumSummaries/queryClusters/msf/Cluster3832.msf) |  |  |  |  |  |  |  |  |  |  |  |  |  |  |
| *nolO* | [3832](http://ccg.murdoch.edu.au/organism/rhizobium/RhizobiumSummaries/queryClusters/msf/Cluster3832.msf) |  |  |  |  |  |  |  |  |  |  |  |  |  |  |
| *nolP* | [17948](http://ccg.murdoch.edu.au/organism/rhizobium/RhizobiumSummaries/queryClusters/msf/Cluster17948.msf) |  |  |  |  |  |  |  |  |  |  |  |  |  |  |
| *nolR* | [5722](http://ccg.murdoch.edu.au/organism/rhizobium/RhizobiumSummaries/queryClusters/msf/Cluster5722.msf) |  |  |  |  |  |  |  |  |  |  |  |  |  |  |
| *nolT* | [9214](http://ccg.murdoch.edu.au/organism/rhizobium/RhizobiumSummaries/queryClusters/msf/Cluster9214.msf) |  |  |  |  |  |  |  |  |  |  |  |  |  |  |
| *nolU* | [6835](http://ccg.murdoch.edu.au/organism/rhizobium/RhizobiumSummaries/queryClusters/msf/Cluster6835.msf) |  |  |  |  |  |  |  |  |  |  |  |  |  |  |

**Table S5.** *Cont.*

| **Coding gene** | **Cluster** | **azc** | **bja** | **bbt** | **bra** | **mlo** | **mes** | **ret** | **rec** | **rlg** | **rlt** | **rle** | **rhi** | **smd** | **sme** |
| --- | --- | --- | --- | --- | --- | --- | --- | --- | --- | --- | --- | --- | --- | --- | --- |
| *nolV* | [6836](http://ccg.murdoch.edu.au/organism/rhizobium/RhizobiumSummaries/queryClusters/msf/Cluster6836.msf) |  |  |  |  |  |  |  |  |  |  |  |  |  |  |
| *nolW* | [5357](http://ccg.murdoch.edu.au/organism/rhizobium/RhizobiumSummaries/queryClusters/msf/Cluster5357.msf) |  |  |  |  |  |  |  |  |  |  |  |  |  |  |
| *nolX* | [9043](http://ccg.murdoch.edu.au/organism/rhizobium/RhizobiumSummaries/queryClusters/msf/Cluster9043.msf) |  |  |  |  |  |  |  |  |  |  |  |  |  |  |
| *nolY* | [12389](http://ccg.murdoch.edu.au/organism/rhizobium/RhizobiumSummaries/queryClusters/seqs/Cluster12389.fa) |  |  |  |  |  |  |  |  |  |  |  |  |  |  |
| *nolZ* | [12388](http://ccg.murdoch.edu.au/organism/rhizobium/RhizobiumSummaries/queryClusters/seqs/Cluster12388.fa) |  |  |  |  |  |  |  |  |  |  |  |  |  |  |

* The gray color indicates absence of the gene.

**Table S6.** Twin arginine transporter system protein orthologs.

| **Coding gene** | **Cluster** | **azc** | **bja** | **bbt** | **bra** | **mlo** | **mes** | **ret** | **rec** | **rlg** | **rlt** | **rle** | **rhi** | **smd** | **sme** |
| --- | --- | --- | --- | --- | --- | --- | --- | --- | --- | --- | --- | --- | --- | --- | --- |
| *tatA* | [10369](http://ccg.murdoch.edu.au/organism/rhizobium/RhizobiumSummaries/queryClusters/seqs/Cluster10369.fa) |  |  |  |  |  |  |  |  |  |  |  |  |  |  |
| *tatA* | [6424](http://ccg.murdoch.edu.au/organism/rhizobium/RhizobiumSummaries/queryClusters/msf/Cluster6424.msf) |  |  |  |  |  |  |  |  |  |  |  |  |  |  |
| *tatA* | [8875](http://ccg.murdoch.edu.au/organism/rhizobium/RhizobiumSummaries/queryClusters/msf/Cluster8875.msf) |  |  |  |  |  |  |  |  |  |  |  |  |  |  |
| *tatA* | [4621](http://ccg.murdoch.edu.au/organism/rhizobium/RhizobiumSummaries/queryClusters/msf/Cluster4621.msf) |  |  |  |  |  |  |  |  |  |  |  |  |  |  |
| *tatA* | [7457](http://ccg.murdoch.edu.au/organism/rhizobium/RhizobiumSummaries/queryClusters/msf/Cluster7457.msf) |  |  |  |  |  |  |  |  |  |  |  |  |  |  |
| *tatA* | [20553](http://ccg.murdoch.edu.au/organism/rhizobium/RhizobiumSummaries/queryClusters/seqs/Cluster20553.fa) |  |  |  |  |  |  |  |  |  |  |  |  |  |  |
| *tatB* | [4991](http://ccg.murdoch.edu.au/organism/rhizobium/RhizobiumSummaries/queryClusters/msf/Cluster4991.msf) |  |  |  |  |  |  |  |  |  |  |  |  |  |  |
| *tatB* | [14080](http://ccg.murdoch.edu.au/organism/rhizobium/RhizobiumSummaries/queryClusters/seqs/Cluster14080.fa) |  |  |  |  |  |  |  |  |  |  |  |  |  |  |
| *tatB* | 14102 |  |  |  |  |  |  |  |  |  |  |  |  |  |  |
| *tatB* | [8115](http://ccg.murdoch.edu.au/organism/rhizobium/RhizobiumSummaries/queryClusters/msf/Cluster8115.msf) |  |  |  |  |  |  |  |  |  |  |  |  |  |  |
| *tatB* | [2731](http://ccg.murdoch.edu.au/organism/rhizobium/RhizobiumSummaries/queryClusters/msf/Cluster2731.msf) |  |  |  |  |  |  |  |  |  |  |  |  |  |  |
| *tatB* | [4770](http://ccg.murdoch.edu.au/organism/rhizobium/RhizobiumSummaries/queryClusters/msf/Cluster4770.msf) |  |  |  |  |  |  |  |  |  |  |  |  |  |  |
| *tatC* | [2980](http://ccg.murdoch.edu.au/organism/rhizobium/RhizobiumSummaries/queryClusters/msf/Cluster2980.msf) |  |  |  |  |  |  |  |  |  |  |  |  |  |  |

* The gray color indicates absence of the gene.

**Table S7.** Type I secretion protein orthologs.

| **Coding gene** | **Cluster** | **azc** | **bja** | **bbt** | **bra** | **mlo** | **mes** | **ret** | **rec** | **rlg** | **rlt** | **rle** | **rhi** | **smd** | **sme** |
| --- | --- | --- | --- | --- | --- | --- | --- | --- | --- | --- | --- | --- | --- | --- | --- |
| *tolC* | [1327](http://ccg.murdoch.edu.au/organism/rhizobium/RhizobiumSummaries/queryClusters/msf/Cluster1327.msf) |  |  |  |  |  |  |  |  |  |  |  |  |  |  |
| *hlyD* family | 51 |  |  |  |  |  |  |  |  |  |  |  |  |  |  |
| *hlyD* | [3801](http://ccg.murdoch.edu.au/organism/rhizobium/RhizobiumSummaries/queryClusters/msf/Cluster3801.msf) |  |  |  |  |  |  |  |  |  |  |  |  |  |  |
| *hlyD* | [2353](http://ccg.murdoch.edu.au/organism/rhizobium/RhizobiumSummaries/queryClusters/msf/Cluster2353.msf) |  |  |  |  |  |  |  |  |  |  |  |  |  |  |
| *nodO* | [19055](http://ccg.murdoch.edu.au/organism/rhizobium/RhizobiumSummaries/queryClusters/seqs/Cluster19055.fa) |  |  |  |  |  |  |  |  |  |  |  |  |  |  |
| *hlyB/aprD/aprE/prtDE* system | [13](http://ccg.murdoch.edu.au/organism/rhizobium/RhizobiumSummaries/queryClusters/msf/Cluster13.msf) |  |  |  |  |  |  |  |  |  |  |  |  |  |  |

* The gray color indicates absence of the gene.

**Table S8.** Type II secretion *sec* orthologs.

| **Coding gene** | **Cluster** | **azc** | **bja** | **bbt** | **bra** | **mlo** | **mes** | **ret** | **rec** | **rlg** | **rlt** | **rle** | **rhi** | **smd** | **sme** |
| --- | --- | --- | --- | --- | --- | --- | --- | --- | --- | --- | --- | --- | --- | --- | --- |
| *secA* | [1374](http://ccg.murdoch.edu.au/organism/rhizobium/RhizobiumSummaries/queryClusters/msf/Cluster1374.msf) |  |  |  |  |  |  |  |  |  |  |  |  |  |  |
| *secB* | [1594](http://ccg.murdoch.edu.au/organism/rhizobium/RhizobiumSummaries/queryClusters/msf/Cluster1594.msf) |  |  |  |  |  |  |  |  |  |  |  |  |  |  |
| *secD/F* | [252](http://ccg.murdoch.edu.au/organism/rhizobium/RhizobiumSummaries/queryClusters/msf/Cluster252.msf) |  |  |  |  |  |  |  |  |  |  |  |  |  |  |
| *secE* | [10212](http://ccg.murdoch.edu.au/organism/rhizobium/RhizobiumSummaries/queryClusters/seqs/Cluster10212.fa) |  |  |  |  |  |  |  |  |  |  |  |  |  |  |
| *secE* | [13078](http://ccg.murdoch.edu.au/organism/rhizobium/RhizobiumSummaries/queryClusters/seqs/Cluster13078.fa) |  |  |  |  |  |  |  |  |  |  |  |  |  |  |
| *secE* | [11643](http://ccg.murdoch.edu.au/organism/rhizobium/RhizobiumSummaries/queryClusters/seqs/Cluster11643.fa) |  |  |  |  |  |  |  |  |  |  |  |  |  |  |
| *secE* | [14008](http://ccg.murdoch.edu.au/organism/rhizobium/RhizobiumSummaries/queryClusters/seqs/Cluster14008.fa) |  |  |  |  |  |  |  |  |  |  |  |  |  |  |
| *secE* | [15467](http://ccg.murdoch.edu.au/organism/rhizobium/RhizobiumSummaries/queryClusters/seqs/Cluster15467.fa) |  |  |  |  |  |  |  |  |  |  |  |  |  |  |
| *secE* | [15053](http://ccg.murdoch.edu.au/organism/rhizobium/RhizobiumSummaries/queryClusters/seqs/Cluster15053.fa) |  |  |  |  |  |  |  |  |  |  |  |  |  |  |
| *secE* | [17716](http://ccg.murdoch.edu.au/organism/rhizobium/RhizobiumSummaries/queryClusters/seqs/Cluster17716.fa) |  |  |  |  |  |  |  |  |  |  |  |  |  |  |
| *secE* | [17204](http://ccg.murdoch.edu.au/organism/rhizobium/RhizobiumSummaries/queryClusters/seqs/Cluster17204.fa) |  |  |  |  |  |  |  |  |  |  |  |  |  |  |
| *secE* | [7448](http://ccg.murdoch.edu.au/organism/rhizobium/RhizobiumSummaries/queryClusters/msf/Cluster7448.msf) |  |  |  |  |  |  |  |  |  |  |  |  |  |  |
| *secE* | [19349](http://ccg.murdoch.edu.au/organism/rhizobium/RhizobiumSummaries/queryClusters/seqs/Cluster19349.fa) |  |  |  |  |  |  |  |  |  |  |  |  |  |  |
| *secE* | [20037](http://ccg.murdoch.edu.au/organism/rhizobium/RhizobiumSummaries/queryClusters/seqs/Cluster20037.fa) |  |  |  |  |  |  |  |  |  |  |  |  |  |  |
| *secE* | [18819](http://ccg.murdoch.edu.au/organism/rhizobium/RhizobiumSummaries/queryClusters/seqs/Cluster18819.fa) |  |  |  |  |  |  |  |  |  |  |  |  |  |  |
| *secG* | [5403](http://ccg.murdoch.edu.au/organism/rhizobium/RhizobiumSummaries/queryClusters/msf/Cluster5403.msf) |  |  |  |  |  |  |  |  |  |  |  |  |  |  |
| *secG* | [15542](http://ccg.murdoch.edu.au/organism/rhizobium/RhizobiumSummaries/queryClusters/seqs/Cluster15542.fa) |  |  |  |  |  |  |  |  |  |  |  |  |  |  |

**Table S8.** *Cont.*

| **Coding gene** | **Cluster** | **azc** | **bja** | **bbt** | **bra** | **mlo** | **mes** | **ret** | **rec** | **rlg** | **rlt** | **rle** | **rhi** | **smd** | **sme** |
| --- | --- | --- | --- | --- | --- | --- | --- | --- | --- | --- | --- | --- | --- | --- | --- |
| *secG* | [15040](http://ccg.murdoch.edu.au/organism/rhizobium/RhizobiumSummaries/queryClusters/seqs/Cluster15040.fa) |  |  |  |  |  |  |  |  |  |  |  |  |  |  |
| *secG* | [4637](http://ccg.murdoch.edu.au/organism/rhizobium/RhizobiumSummaries/queryClusters/msf/Cluster4637msf) |  |  |  |  |  |  |  |  |  |  |  |  |  |  |
| *secY* | [1298](http://ccg.murdoch.edu.au/organism/rhizobium/RhizobiumSummaries/queryClusters/msf/Cluster1298.msf) |  |  |  |  |  |  |  |  |  |  |  |  |  |  |
| *yidC* | [1464](http://ccg.murdoch.edu.au/organism/rhizobium/RhizobiumSummaries/queryClusters/msf/Cluster1464.msf) |  |  |  |  |  |  |  |  |  |  |  |  |  |  |
| *ftsY* | [1420](http://ccg.murdoch.edu.au/organism/rhizobium/RhizobiumSummaries/queryClusters/msf/Cluster1420.msf) |  |  |  |  |  |  |  |  |  |  |  |  |  |  |
| *ffh* | [1439](http://ccg.murdoch.edu.au/organism/rhizobium/RhizobiumSummaries/queryClusters/msf/Cluster1439.msf) |  |  |  |  |  |  |  |  |  |  |  |  |  |  |
| *yacJ* | [1344](http://ccg.murdoch.edu.au/organism/rhizobium/RhizobiumSummaries/queryClusters/msf/Cluster1344.msf) |  |  |  |  |  |  |  |  |  |  |  |  |  |  |

* The gray color indicates absence of the gene.

**Table S9.** Type II secretion gsp orthologs.

| **Coding gene** | **Cluster** | **azc** | **bja** | **bbt** | **bra** | **mlo** | **mes** | **ret** | **rec** | **rlg** | **rlt** | **rle** | **rhi** | **smd** | **sme** |
| --- | --- | --- | --- | --- | --- | --- | --- | --- | --- | --- | --- | --- | --- | --- | --- |
| *gspD* | [3007](http://ccg.murdoch.edu.au/organism/rhizobium/RhizobiumSummaries/queryClusters/msf/Cluster3007.msf) |  |  |  |  |  |  |  |  |  |  |  |  |  |  |
| *gspE* | [3382](http://ccg.murdoch.edu.au/organism/rhizobium/RhizobiumSummaries/queryClusters/msf/Cluster3382.msf) |  |  |  |  |  |  |  |  |  |  |  |  |  |  |
| *gspF* | [9051](http://ccg.murdoch.edu.au/organism/rhizobium/RhizobiumSummaries/queryClusters/msf/Cluster9051msf) |  |  |  |  |  |  |  |  |  |  |  |  |  |  |
| *gspF* | [12992](http://ccg.murdoch.edu.au/organism/rhizobium/RhizobiumSummaries/queryClusters/seqs/Cluster12992.fa) |  |  |  |  |  |  |  |  |  |  |  |  |  |  |
| *gspF* | [4258](http://ccg.murdoch.edu.au/organism/rhizobium/RhizobiumSummaries/queryClusters/msf/Cluster4258.msf) |  |  |  |  |  |  |  |  |  |  |  |  |  |  |
| *gspG* | [3383](http://ccg.murdoch.edu.au/organism/rhizobium/RhizobiumSummaries/queryClusters/msf/Cluster3383.msf) |  |  |  |  |  |  |  |  |  |  |  |  |  |  |
| *gspH* | [16602](http://ccg.murdoch.edu.au/organism/rhizobium/RhizobiumSummaries/queryClusters/seqs/Cluster16602.fa) |  |  |  |  |  |  |  |  |  |  |  |  |  |  |
| *gspH* | [18602](http://ccg.murdoch.edu.au/organism/rhizobium/RhizobiumSummaries/queryClusters/seqs/Cluster18602.fa) |  |  |  |  |  |  |  |  |  |  |  |  |  |  |
| *gspH* | [3750](http://ccg.murdoch.edu.au/organism/rhizobium/RhizobiumSummaries/queryClusters/msf/Cluster3750.msf) |  |  |  |  |  |  |  |  |  |  |  |  |  |  |
| *gspI* | [16603](http://ccg.murdoch.edu.au/organism/rhizobium/RhizobiumSummaries/queryClusters/seqs/Cluster16603.fa) |  |  |  |  |  |  |  |  |  |  |  |  |  |  |
| *gspI* | [18601](http://ccg.murdoch.edu.au/organism/rhizobium/RhizobiumSummaries/queryClusters/seqs/Cluster18601.fa) |  |  |  |  |  |  |  |  |  |  |  |  |  |  |
| *gspI* | [13907](http://ccg.murdoch.edu.au/organism/rhizobium/RhizobiumSummaries/queryClusters/seqs/Cluster13907.fa) |  |  |  |  |  |  |  |  |  |  |  |  |  |  |
| *gspI* | [7996](http://ccg.murdoch.edu.au/organism/rhizobium/RhizobiumSummaries/queryClusters/msf/Cluster7996.msf) |  |  |  |  |  |  |  |  |  |  |  |  |  |  |
| *gspJ* | [16604](http://ccg.murdoch.edu.au/organism/rhizobium/RhizobiumSummaries/queryClusters/seqs/Cluster16604.fa) |  |  |  |  |  |  |  |  |  |  |  |  |  |  |
| *gspJ* | [18600](http://ccg.murdoch.edu.au/organism/rhizobium/RhizobiumSummaries/queryClusters/seqs/Cluster18600.fa) |  |  |  |  |  |  |  |  |  |  |  |  |  |  |
| *gspJ* | [3751](http://ccg.murdoch.edu.au/organism/rhizobium/RhizobiumSummaries/queryClusters/msf/Cluster3751.msf) |  |  |  |  |  |  |  |  |  |  |  |  |  |  |

**Table S9.** *Cont.*

| **Coding gene** | **Cluster** | **azc** | **bja** | **bbt** | **bra** | **mlo** | **mes** | **ret** | **rec** | **rlg** | **rlt** | **rle** | **rhi** | **smd** | **sme** |
| --- | --- | --- | --- | --- | --- | --- | --- | --- | --- | --- | --- | --- | --- | --- | --- |
| *gspK* | [16601](http://ccg.murdoch.edu.au/organism/rhizobium/RhizobiumSummaries/queryClusters/seqs/Cluster16601.fa) |  |  |  |  |  |  |  |  |  |  |  |  |  |  |
| *gspK* | [18603](http://ccg.murdoch.edu.au/organism/rhizobium/RhizobiumSummaries/queryClusters/seqs/Cluster18603.fa) |  |  |  |  |  |  |  |  |  |  |  |  |  |  |
| *gspK* | [3752](http://ccg.murdoch.edu.au/organism/rhizobium/RhizobiumSummaries/queryClusters/msf/Cluster3752.msf) |  |  |  |  |  |  |  |  |  |  |  |  |  |  |
| *gspL* | [9050](http://ccg.murdoch.edu.au/organism/rhizobium/RhizobiumSummaries/queryClusters/msf/Cluster9050.msf) |  |  |  |  |  |  |  |  |  |  |  |  |  |  |
| *gspL* | [4259](http://ccg.murdoch.edu.au/organism/rhizobium/RhizobiumSummaries/queryClusters/msf/Cluster4259.msf) |  |  |  |  |  |  |  |  |  |  |  |  |  |  |
| *gspM* | [4260](http://ccg.murdoch.edu.au/organism/rhizobium/RhizobiumSummaries/queryClusters/msf/Cluster4260.msf) |  |  |  |  |  |  |  |  |  |  |  |  |  |  |

* The gray color indicates absence of the gene.

**Table S10.** Type III secretion *nop/ysc/rhc* orthologs.

| **Coding gene** | **Cluster** | **azc** | **bja** | **bbt** | **bra** | **mlo** | **mes** | **ret** | **rec** | **rlg** | **rlt** | **rle** | **rhi** | **smd** | **sme** |
| --- | --- | --- | --- | --- | --- | --- | --- | --- | --- | --- | --- | --- | --- | --- | --- |
| *nopL* | [18019](http://ccg.murdoch.edu.au/organism/rhizobium/RhizobiumSummaries/queryClusters/seqs/Cluster18019.fa) |  |  |  |  |  |  |  |  |  |  |  |  |  |  |
| *nopP* | [4327](http://ccg.murdoch.edu.au/organism/rhizobium/RhizobiumSummaries/queryClusters/msf/Cluster4327.msf) |  |  |  |  |  |  |  |  |  |  |  |  |  |  |
| *nopT* | [6843](http://ccg.murdoch.edu.au/organism/rhizobium/RhizobiumSummaries/queryClusters/msf/Cluster6843msf) |  |  |  |  |  |  |  |  |  |  |  |  |  |  |
| *nopJ* | [17997](http://ccg.murdoch.edu.au/organism/rhizobium/RhizobiumSummaries/queryClusters/seqs/Cluster17997.fa) |  |  |  |  |  |  |  |  |  |  |  |  |  |  |
| *nopM* | [4356](http://ccg.murdoch.edu.au/organism/rhizobium/RhizobiumSummaries/queryClusters/msf/Cluster4356.msf) |  |  |  |  |  |  |  |  |  |  |  |  |  |  |
| *yscC/rhcC* | [5357](http://ccg.murdoch.edu.au/organism/rhizobium/RhizobiumSummaries/queryClusters/msf/Cluster5357.msf) |  |  |  |  |  |  |  |  |  |  |  |  |  |  |
| *yscJ/rhcJ* | [4358](http://ccg.murdoch.edu.au/organism/rhizobium/RhizobiumSummaries/queryClusters/msf/Cluster4358.msf) |  |  |  |  |  |  |  |  |  |  |  |  |  |  |
| *yscJ/rhcJ* | [9214](http://ccg.murdoch.edu.au/organism/rhizobium/RhizobiumSummaries/queryClusters/msf/Cluster9214.msf) |  |  |  |  |  |  |  |  |  |  |  |  |  |  |
| *yscS/rhcS* | [9045](http://ccg.murdoch.edu.au/organism/rhizobium/RhizobiumSummaries/queryClusters/msf/Cluster9045.msf) |  |  |  |  |  |  |  |  |  |  |  |  |  |  |
| *yscS/rhcS* | [14734](http://ccg.murdoch.edu.au/organism/rhizobium/RhizobiumSummaries/queryClusters/seqs/Cluster14734.fa) |  |  |  |  |  |  |  |  |  |  |  |  |  |  |
| *yscS/rhcS* | [12305](http://ccg.murdoch.edu.au/organism/rhizobium/RhizobiumSummaries/queryClusters/seqs/Cluster12305.fa) |  |  |  |  |  |  |  |  |  |  |  |  |  |  |
| *yscR/rhcR/fliP* | [371](http://ccg.murdoch.edu.au/organism/rhizobium/RhizobiumSummaries/queryClusters/msf/Cluster371.msf) |  |  |  |  |  |  |  |  |  |  |  |  |  |  |
| *yscT/rhcT* | [5358](http://ccg.murdoch.edu.au/organism/rhizobium/RhizobiumSummaries/queryClusters/msf/Cluster5358.msf) |  |  |  |  |  |  |  |  |  |  |  |  |  |  |
| *yscT/rhcT* | [14735](http://ccg.murdoch.edu.au/organism/rhizobium/RhizobiumSummaries/queryClusters/seqs/Cluster14735.fa) |  |  |  |  |  |  |  |  |  |  |  |  |  |  |
| *yscT/rhcT* | [9218](http://ccg.murdoch.edu.au/organism/rhizobium/RhizobiumSummaries/queryClusters/msf/Cluster9218.msf) |  |  |  |  |  |  |  |  |  |  |  |  |  |  |
| *yscU/rhcU* | [5359](http://ccg.murdoch.edu.au/organism/rhizobium/RhizobiumSummaries/queryClusters/msf/Cluster5359.msf) |  |  |  |  |  |  |  |  |  |  |  |  |  |  |
| *yscU/rhcU* | [9219](http://ccg.murdoch.edu.au/organism/rhizobium/RhizobiumSummaries/queryClusters/msf/Cluster9219.msf) |  |  |  |  |  |  |  |  |  |  |  |  |  |  |

**Table S10.** *Cont.*

| **Coding gene** | **Cluster** | **azc** | **bja** | **bbt** | **bra** | **mlo** | **mes** | **ret** | **rec** | **rlg** | **rlt** | **rle** | **rhi** | **smd** | **sme** |
| --- | --- | --- | --- | --- | --- | --- | --- | --- | --- | --- | --- | --- | --- | --- | --- |
| *yscVrhcV/flhA *** | [373](http://ccg.murdoch.edu.au/organism/rhizobium/RhizobiumSummaries/queryClusters/msf/Cluster373.msf) |  |  |  |  |  |  |  |  |  |  |  |  |  |  |
| *yscN/rhcN/fliI**** | [372](http://ccg.murdoch.edu.au/organism/rhizobium/RhizobiumSummaries/queryClusters/msf/Cluster372.msf) |  |  |  |  |  |  |  |  |  |  |  |  |  |  |
| *yscQ/rhcQ/fliN* | [6838](http://ccg.murdoch.edu.au/organism/rhizobium/RhizobiumSummaries/queryClusters/msf/Cluster6838.msf) |  |  |  |  |  |  |  |  |  |  |  |  |  |  |
| *yscQ/rhcQ/fliN* | [9217](http://ccg.murdoch.edu.au/organism/rhizobium/RhizobiumSummaries/queryClusters/msf/Cluster9217.msf) |  |  |  |  |  |  |  |  |  |  |  |  |  |  |
| *yscL/nolV* | [6836](http://ccg.murdoch.edu.au/organism/rhizobium/RhizobiumSummaries/queryClusters/msf/Cluster6836.msf) |  |  |  |  |  |  |  |  |  |  |  |  |  |  |
| *yscL/nolV* | [9216](http://ccg.murdoch.edu.au/organism/rhizobium/RhizobiumSummaries/queryClusters/msf/Cluster9216.msf) |  |  |  |  |  |  |  |  |  |  |  |  |  |  |

* The gray color indicates absence of the gene.

**Table S11.** F-type Type IV secretion protein orthologs.

| **Coding gene** | **Cluster** | **azc** | **bja** | **bbt** | **bra** | **mlo** | **mes** | **ret** | **rec** | **rlg** | **rlt** | **rle** | **rhi** | **smd** | **sme** |
| --- | --- | --- | --- | --- | --- | --- | --- | --- | --- | --- | --- | --- | --- | --- | --- |
| *trbD* | 2283 |  |  |  |  |  |  |  |  |  |  |  |  |  |  |
| *trbJ* | 2105 |  |  |  |  |  |  |  |  |  |  |  |  |  |  |
| *trbL* | 1194 |  |  |  |  |  |  |  |  |  |  |  |  |  |  |
| *trbF* | 1195 |  |  |  |  |  |  |  |  |  |  |  |  |  |  |
| *trbI* | 572 |  |  |  |  |  |  |  |  |  |  |  |  |  |  |
| *virB1* | 3079 |  |  |  |  |  |  |  |  |  |  |  |  |  |  |
| *tIrB2/trbC* | 5995 |  |  |  |  |  |  |  |  |  |  |  |  |  |  |
| *tIrB2/trbC* | 13648 |  |  |  |  |  |  |  |  |  |  |  |  |  |  |
| *tIrB2/trbC* | 9347 |  |  |  |  |  |  |  |  |  |  |  |  |  |  |
| *tIrB2/trbC* | 5706 |  |  |  |  |  |  |  |  |  |  |  |  |  |  |
| *tIrB2/trbC* | 10779 |  |  |  |  |  |  |  |  |  |  |  |  |  |  |
| *virB3* | 3365 |  |  |  |  |  |  |  |  |  |  |  |  |  |  |
| *virB4* | 9336 |  |  |  |  |  |  |  |  |  |  |  |  |  |  |
| *virB4/TrbE* | 1621 |  |  |  |  |  |  |  |  |  |  |  |  |  |  |
| *virB4/TrbE* | 647 |  |  |  |  |  |  |  |  |  |  |  |  |  |  |
| *virB5* | 3364 |  |  |  |  |  |  |  |  |  |  |  |  |  |  |
| *virB5* | 9337 |  |  |  |  |  |  |  |  |  |  |  |  |  |  |

**Table S11.** *Cont.*

| **Coding gene** | **Cluster** | **azc** | **bja** | **bbt** | **bra** | **mlo** | **mes** | **ret** | **rec** | **rlg** | **rlt** | **rle** | **rhi** | **smd** | **sme** |
| --- | --- | --- | --- | --- | --- | --- | --- | --- | --- | --- | --- | --- | --- | --- | --- |
| *virB5* | 19707 |  |  |  |  |  |  |  |  |  |  |  |  |  |  |
| *virB6/trbL* | 3363 |  |  |  |  |  |  |  |  |  |  |  |  |  |  |
| *virB6/trbL* | 20231 |  |  |  |  |  |  |  |  |  |  |  |  |  |  |
| *virB6/trbL* | 4866 |  |  |  |  |  |  |  |  |  |  |  |  |  |  |
| *VirB7* | 18203 |  |  |  |  |  |  |  |  |  |  |  |  |  |  |
| *VirB7* | 17844 |  |  |  |  |  |  |  |  |  |  |  |  |  |  |
| *VirB7* | 7219 |  |  |  |  |  |  |  |  |  |  |  |  |  |  |
| *virB8/trbF* | 2126 |  |  |  |  |  |  |  |  |  |  |  |  |  |  |
| *virB8/trbF* | 9339 |  |  |  |  |  |  |  |  |  |  |  |  |  |  |
| *virB8/trbF* | 4865 |  |  |  |  |  |  |  |  |  |  |  |  |  |  |
| *VirB9/TrbG* | 2296 |  |  |  |  |  |  |  |  |  |  |  |  |  |  |
| *VirB9/TrbG* | 5412 |  |  |  |  |  |  |  |  |  |  |  |  |  |  |
| *VirB9/TrbG* | 648 |  |  |  |  |  |  |  |  |  |  |  |  |  |  |
| *VirB10/TrbI* | 1620 |  |  |  |  |  |  |  |  |  |  |  |  |  |  |
| *VirB11/TrbB* | 58 |  |  |  |  |  |  |  |  |  |  |  |  |  |  |

* The gray color indicates absence of the gene.

**Table S12.** P-type type IV secretion protein orthologs.

| **Coding gene** | **Cluster** | **azc** | **bja** | **bbt** | **bra** | **mlo** | **mes** | **ret** | **rec** | **rlg** | **rlt** | **rle** | **rhi** | **smd** | **sme** |
| --- | --- | --- | --- | --- | --- | --- | --- | --- | --- | --- | --- | --- | --- | --- | --- |
| *cpaA* | [2527](http://ccg.murdoch.edu.au/organism/rhizobium/RhizobiumSummaries/queryClusters/msf/Cluster2527.msf) |  |  |  |  |  |  |  |  |  |  |  |  |  |  |
| *cpaB* | [464](http://ccg.murdoch.edu.au/organism/rhizobium/RhizobiumSummaries/queryClusters/msf/Cluster464.msf) |  |  |  |  |  |  |  |  |  |  |  |  |  |  |
| *cpaC* | [179](http://ccg.murdoch.edu.au/organism/rhizobium/RhizobiumSummaries/queryClusters/msf/Cluster179.msf) |  |  |  |  |  |  |  |  |  |  |  |  |  |  |
| *cpaD* | [779](http://ccg.murdoch.edu.au/organism/rhizobium/RhizobiumSummaries/queryClusters/msf/Cluster779.msf) |  |  |  |  |  |  |  |  |  |  |  |  |  |  |
| *cpaE* | [397](http://ccg.murdoch.edu.au/organism/rhizobium/RhizobiumSummaries/queryClusters/msf/Cluster397.msf) |  |  |  |  |  |  |  |  |  |  |  |  |  |  |
| *cpaF* | [58](http://ccg.murdoch.edu.au/organism/rhizobium/RhizobiumSummaries/queryClusters/msf/Cluster58.msf) |  |  |  |  |  |  |  |  |  |  |  |  |  |  |
| *tadB* | [358](http://ccg.murdoch.edu.au/organism/rhizobium/RhizobiumSummaries/queryClusters/msf/Cluster358.msf) |  |  |  |  |  |  |  |  |  |  |  |  |  |  |
| *tadC* | [461](http://ccg.murdoch.edu.au/organism/rhizobium/RhizobiumSummaries/queryClusters/msf/Cluster462.msf) |  |  |  |  |  |  |  |  |  |  |  |  |  |  |

**Table S12.** *Cont.*

| **Coding gene** | **Cluster** | **azc** | **bja** | **bbt** | **bra** | **mlo** | **mes** | **ret** | **rec** | **rlg** | **rlt** | **rle** | **rhi** | **smd** | **sme** |
| --- | --- | --- | --- | --- | --- | --- | --- | --- | --- | --- | --- | --- | --- | --- | --- |
| *pilA* | 4511 |  |  |  |  |  |  |  |  |  |  |  |  |  |  |
| *pilA* | [18209](http://ccg.murdoch.edu.au/organism/rhizobium/RhizobiumSummaries/queryClusters/seqs/Cluster182092.msf) |  |  |  |  |  |  |  |  |  |  |  |  |  |  |

* The gray color indicates absence of the gene.

**Table S13.** Type V secretion protein orthologs.

| **Coding gene** | **Cluster** | **azc** | **bja** | **bbt** | **bra** | **mlo** | **mes** | **ret** | **rec** | **rlg** | **rlt** | **rle** | **rhi** | **smd** | **sme** |
| --- | --- | --- | --- | --- | --- | --- | --- | --- | --- | --- | --- | --- | --- | --- | --- |
| *autA* | [3479](http://ccg.murdoch.edu.au/organism/rhizobium/RhizobiumSummaries/queryClusters/msf/Cluster3479.msf) |  |  |  |  |  |  |  |  |  |  |  |  |  |  |
| *autB* | [3479](http://ccg.murdoch.edu.au/organism/rhizobium/RhizobiumSummaries/queryClusters/msf/Cluster3479.msf) |  |  |  |  |  |  |  |  |  |  |  |  |  |  |
| *autC* | [599](http://ccg.murdoch.edu.au/organism/rhizobium/RhizobiumSummaries/queryClusters/msf/Cluster599.msf) |  |  |  |  |  |  |  |  |  |  |  |  |  |  |

* The gray color indicates absence of the gene.

**Table S14.** Type VI secretion protein orthologs.

| **Coding gene** | **Cluster** | **azc** | **bja** | **bbt** | **bra** | **mlo** | **mes** | **ret** | **rec** | **rlg** | **rlt** | **rle** | **rhi** | **smd** | **sme** |
| --- | --- | --- | --- | --- | --- | --- | --- | --- | --- | --- | --- | --- | --- | --- | --- |
| *impA* | 7826 |  |  |  |  |  |  |  |  |  |  |  |  |  |  |
| *impB* | 3667 |  |  |  |  |  |  |  |  |  |  |  |  |  |  |
| *impC* | 3479 |  |  |  |  |  |  |  |  |  |  |  |  |  |  |
| *impD* | 2956 |  |  |  |  |  |  |  |  |  |  |  |  |  |  |
| *impE* | 7827 |  |  |  |  |  |  |  |  |  |  |  |  |  |  |
| *impF* | 18699 |  |  |  |  |  |  |  |  |  |  |  |  |  |  |
| *impG* | 3331 |  |  |  |  |  |  |  |  |  |  |  |  |  |  |
| *impH* | 3668 |  |  |  |  |  |  |  |  |  |  |  |  |  |  |
| *impI* | 18698 |  |  |  |  |  |  |  |  |  |  |  |  |  |  |
| *impJ* | 3666 |  |  |  |  |  |  |  |  |  |  |  |  |  |  |
| *impK* | 3329 |  |  |  |  |  |  |  |  |  |  |  |  |  |  |
| *impL* | 3330 |  |  |  |  |  |  |  |  |  |  |  |  |  |  |
| *impM* | 18697 |  |  |  |  |  |  |  |  |  |  |  |  |  |  |
| *impN* | 18696 |  |  |  |  |  |  |  |  |  |  |  |  |  |  |

* The gray color indicates absence of the gene.

**Table S15.** Exo exopolysacchride synthesis protein orthologs.

| **Coding gene** | **Cluster** | **azc** | **bja** | **bbt** | **bra** | **mlo** | **mes** | **ret** | **rec** | **rlg** | **rlt** | **rle** | **rhi** | **smd** | **sme** |
| --- | --- | --- | --- | --- | --- | --- | --- | --- | --- | --- | --- | --- | --- | --- | --- |
| *exoA* | 3887 |  |  |  |  |  |  |  |  |  |  |  |  |  |  |
| *exoA* | 103 |  |  |  |  |  |  |  |  |  |  |  |  |  |  |
| *exoB* | 130 |  |  |  |  |  |  |  |  |  |  |  |  |  |  |
| *exoD* | 5463 |  |  |  |  |  |  |  |  |  |  |  |  |  |  |
| *exoD* | 2952 |  |  |  |  |  |  |  |  |  |  |  |  |  |  |
| *exoF* | 2720 |  |  |  |  |  |  |  |  |  |  |  |  |  |  |
| *exoH* | 18229 |  |  |  |  |  |  |  |  |  |  |  |  |  |  |
| *exoI* | 2139 |  |  |  |  |  |  |  |  |  |  |  |  |  |  |
| *exoI* | 1865 |  |  |  |  |  |  |  |  |  |  |  |  |  |  |
| *exoL* | 4424 |  |  |  |  |  |  |  |  |  |  |  |  |  |  |
| *exoM* | 4425 |  |  |  |  |  |  |  |  |  |  |  |  |  |  |
| *exoM/pssC* | 4707 |  |  |  |  |  |  |  |  |  |  |  |  |  |  |
| *exoN* | 474 |  |  |  |  |  |  |  |  |  |  |  |  |  |  |
| *exoO* | 3480 |  |  |  |  |  |  |  |  |  |  |  |  |  |  |
| *exoP/pssP* | 357 |  |  |  |  |  |  |  |  |  |  |  |  |  |  |
| *exoP* | 1805 |  |  |  |  |  |  |  |  |  |  |  |  |  |  |
| *exoQ* | 4423 |  |  |  |  |  |  |  |  |  |  |  |  |  |  |
| *exoQ* | 4795 |  |  |  |  |  |  |  |  |  |  |  |  |  |  |
| *exoR* | 861 |  |  |  |  |  |  |  |  |  |  |  |  |  |  |
| *exoS* | 1469 |  |  |  |  |  |  |  |  |  |  |  |  |  |  |
| *exoT* | 9669 |  |  |  |  |  |  |  |  |  |  |  |  |  |  |
| *exoT* | 4422 |  |  |  |  |  |  |  |  |  |  |  |  |  |  |
| *exoU* | 4482 |  |  |  |  |  |  |  |  |  |  |  |  |  |  |
| *exoU* | 3496 |  |  |  |  |  |  |  |  |  |  |  |  |  |  |
| *exoV/pssM* | 3083 |  |  |  |  |  |  |  |  |  |  |  |  |  |  |
| *exoX* | 9679 |  |  |  |  |  |  |  |  |  |  |  |  |  |  |

**Table S15.** *Cont.*

| **Coding gene** | **Cluster** | **azc** | **bja** | **bbt** | **bra** | **mlo** | **mes** | **ret** | **rec** | **rlg** | **rlt** | **rle** | **rhi** | **smd** | **sme** |
| --- | --- | --- | --- | --- | --- | --- | --- | --- | --- | --- | --- | --- | --- | --- | --- |
| *exoX* | 9248 |  |  |  |  |  |  |  |  |  |  |  |  |  |  |
| *exoY1* | 105 |  |  |  |  |  |  |  |  |  |  |  |  |  |  |
| *exoY2* | 520 |  |  |  |  |  |  |  |  |  |  |  |  |  |  |
| *exoZ1* | 1768 |  |  |  |  |  |  |  |  |  |  |  |  |  |  |
| *exoZ2/exoK* | 4421 |  |  |  |  |  |  |  |  |  |  |  |  |  |  |

* The gray color indicates absence of the gene.

**Table S16.** Pss exopolysacchride synthesis protein orthologs.

| **Coding gene** | **Cluster** | **azc** | **bja** | **bbt** | **bra** | **mlo** | **mes** | **ret** | **rec** | **rlg** | **rlt** | **rle** | **rhi** | **smd** | **sme** |
| --- | --- | --- | --- | --- | --- | --- | --- | --- | --- | --- | --- | --- | --- | --- | --- |
| *pssA* | 1160 |  |  |  |  |  |  |  |  |  |  |  |  |  |  |
| *pssB* | 615 |  |  |  |  |  |  |  |  |  |  |  |  |  |  |
| *pssC* | 4707 |  |  |  |  |  |  |  |  |  |  |  |  |  |  |
| *pssD* | 4709 |  |  |  |  |  |  |  |  |  |  |  |  |  |  |
| *pssE* | 4710 |  |  |  |  |  |  |  |  |  |  |  |  |  |  |
| *pssF* | 4706 |  |  |  |  |  |  |  |  |  |  |  |  |  |  |
| *pssF* | 7396 |  |  |  |  |  |  |  |  |  |  |  |  |  |  |
| *pssG* | 2557 |  |  |  |  |  |  |  |  |  |  |  |  |  |  |
| *pssH* | 2557 |  |  |  |  |  |  |  |  |  |  |  |  |  |  |
| *pssI* | 2557 |  |  |  |  |  |  |  |  |  |  |  |  |  |  |
| *pssJ* | 5772 |  |  |  |  |  |  |  |  |  |  |  |  |  |  |
| *pssK* | 4082 |  |  |  |  |  |  |  |  |  |  |  |  |  |  |
| *pssL* | 4705 |  |  |  |  |  |  |  |  |  |  |  |  |  |  |
| *pssM/exoV* | 3083 |  |  |  |  |  |  |  |  |  |  |  |  |  |  |
| *pssN/Wza* | 678 |  |  |  |  |  |  |  |  |  |  |  |  |  |  |
| *pssO* | 4713 |  |  |  |  |  |  |  |  |  |  |  |  |  |  |
| *pssP/exoP* | 357 |  |  |  |  |  |  |  |  |  |  |  |  |  |  |

**Table S16.** *Cont.*

| **Coding gene** | **Cluster** | **azc** | **bja** | **bbt** | **bra** | **mlo** | **mes** | **ret** | **rec** | **rlg** | **rlt** | **rle** | **rhi** | **smd** | **sme** |
| --- | --- | --- | --- | --- | --- | --- | --- | --- | --- | --- | --- | --- | --- | --- | --- |
| *pssR* | 4704 |  |  |  |  |  |  |  |  |  |  |  |  |  |  |
| *pssS* | 3579 |  |  |  |  |  |  |  |  |  |  |  |  |  |  |
| *pssT/Wzy* | 2644 |  |  |  |  |  |  |  |  |  |  |  |  |  |  |
| *pssV* | 2891 |  |  |  |  |  |  |  |  |  |  |  |  |  |  |

**Table S17.** Fix orthologs.

| **Coding gene** | **Cluster** | **azc** | **bja** | **bbt** | **bra** | **mlo** | **mes** | **ret** | **rec** | **rlg** | **rlt** | **rle** | **rhi** | **smd** | **sme** |
| --- | --- | --- | --- | --- | --- | --- | --- | --- | --- | --- | --- | --- | --- | --- | --- |
| *fixA* | [1726](http://ccg.murdoch.edu.au/organism/rhizobium/RhizobiumSummaries/queryClusters/msf/Cluster1726.msf) |  |  |  |  |  |  |  |  |  |  |  |  |  |  |
| *fixB* | [1727](http://ccg.murdoch.edu.au/organism/rhizobium/RhizobiumSummaries/queryClusters/msf/Cluster1727.msf) |  |  |  |  |  |  |  |  |  |  |  |  |  |  |
| *fixC* | [1728](http://ccg.murdoch.edu.au/organism/rhizobium/RhizobiumSummaries/queryClusters/msf/Cluster1728.msf) |  |  |  |  |  |  |  |  |  |  |  |  |  |  |
| *fixX* | [1939](http://ccg.murdoch.edu.au/organism/rhizobium/RhizobiumSummaries/queryClusters/msf/Cluster1939.msf) |  |  |  |  |  |  |  |  |  |  |  |  |  |  |
| *fixF* | [17970](http://ccg.murdoch.edu.au/organism/rhizobium/RhizobiumSummaries/queryClusters/seqs/Cluster17970fa) |  |  |  |  |  |  |  |  |  |  |  |  |  |  |
| *fixJ* | [63](http://ccg.murdoch.edu.au/organism/rhizobium/RhizobiumSummaries/queryClusters/msf/Cluster63.msf) |  |  |  |  |  |  |  |  |  |  |  |  |  |  |
| *fixK* | [420](http://ccg.murdoch.edu.au/organism/rhizobium/RhizobiumSummaries/queryClusters/msf/Cluster420.msf) |  |  |  |  |  |  |  |  |  |  |  |  |  |  |
| *fixL* | [82](http://ccg.murdoch.edu.au/organism/rhizobium/RhizobiumSummaries/queryClusters/msf/Cluster82.msf) |  |  |  |  |  |  |  |  |  |  |  |  |  |  |
| *fixM* | [676](http://ccg.murdoch.edu.au/organism/rhizobium/RhizobiumSummaries/queryClusters/msf/Cluster676.msf) |  |  |  |  |  |  |  |  |  |  |  |  |  |  |
| *fixN* | [350](http://ccg.murdoch.edu.au/organism/rhizobium/RhizobiumSummaries/queryClusters/msf/Cluster350.msf) |  |  |  |  |  |  |  |  |  |  |  |  |  |  |
| *fixO* | [351](http://ccg.murdoch.edu.au/organism/rhizobium/RhizobiumSummaries/queryClusters/msf/Cluster351.msf) |  |  |  |  |  |  |  |  |  |  |  |  |  |  |
| *fixP* | [352](http://ccg.murdoch.edu.au/organism/rhizobium/RhizobiumSummaries/queryClusters/msf/Cluster352.msf) |  |  |  |  |  |  |  |  |  |  |  |  |  |  |
| *fixQ* | [18756](http://ccg.murdoch.edu.au/organism/rhizobium/RhizobiumSummaries/queryClusters/seqs/Cluster18756.fa) |  |  |  |  |  |  |  |  |  |  |  |  |  |  |
| *fixQ* | [18725](http://ccg.murdoch.edu.au/organism/rhizobium/RhizobiumSummaries/queryClusters/seqs/Cluster18725.msf) |  |  |  |  |  |  |  |  |  |  |  |  |  |  |
| *fixQ* | [7676](http://ccg.murdoch.edu.au/organism/rhizobium/RhizobiumSummaries/queryClusters/msf/Cluster7676.msf) |  |  |  |  |  |  |  |  |  |  |  |  |  |  |
| *fixQ* | [7256](http://ccg.murdoch.edu.au/organism/rhizobium/RhizobiumSummaries/queryClusters/msf/Cluster7256.msf) |  |  |  |  |  |  |  |  |  |  |  |  |  |  |
| *fixQ* | [12558](http://ccg.murdoch.edu.au/organism/rhizobium/RhizobiumSummaries/queryClusters/seqs/Cluster12558.fa) |  |  |  |  |  |  |  |  |  |  |  |  |  |  |
| *fixQ* | [13950](http://ccg.murdoch.edu.au/organism/rhizobium/RhizobiumSummaries/queryClusters/seqs/Cluster13950.fa) |  |  |  |  |  |  |  |  |  |  |  |  |  |  |
| *fixQ* | [7222](http://ccg.murdoch.edu.au/organism/rhizobium/RhizobiumSummaries/queryClusters/msf/Cluster7222.msf) |  |  |  |  |  |  |  |  |  |  |  |  |  |  |
| *fixQ* | [16567](http://ccg.murdoch.edu.au/organism/rhizobium/RhizobiumSummaries/queryClusters/seqs/Cluster16567.fa) |  |  |  |  |  |  |  |  |  |  |  |  |  |  |

**Table S17.** *Cont.*

| **Coding gene** | | **Cluster** | **azc** | **bja** | **bbt** | **bra** | **mlo** | **mes** | **ret** | **rec** | **rlg** | **rlt** | **rle** | **rhi** | | **smd** | **sme** |
| --- | --- | --- | --- | --- | --- | --- | --- | --- | --- | --- | --- | --- | --- | --- | --- | --- | --- |
| *fixQ* | | [16510](http://ccg.murdoch.edu.au/organism/rhizobium/RhizobiumSummaries/queryClusters/seqs/Cluster16510.fa) |  |  |  |  |  |  |  |  |  |  |  |  | |  |  |
| *fixQ* | | [10911](http://ccg.murdoch.edu.au/organism/rhizobium/RhizobiumSummaries/queryClusters/seqs/Cluster10911.fa) |  |  |  |  |  |  |  |  |  |  |  |  | |  |  |
| *fixR* | | [2241](http://ccg.murdoch.edu.au/organism/rhizobium/RhizobiumSummaries/queryClusters/msf/Cluster2241.msf) |  |  |  |  |  |  |  |  |  |  |  |  | |  |  |
| *fixG* | | [394](http://ccg.murdoch.edu.au/organism/rhizobium/RhizobiumSummaries/queryClusters/msf/Cluster394.msf) |  |  |  |  |  |  |  |  |  |  |  |  | |  |  |
| *fixH* | | [5105](http://ccg.murdoch.edu.au/organism/rhizobium/RhizobiumSummaries/queryClusters/msf/Cluster5105.msf) |  |  |  |  |  |  |  |  |  |  |  |  | |  |  |
| *fixH* | | [2028](http://ccg.murdoch.edu.au/organism/rhizobium/RhizobiumSummaries/queryClusters/msf/Cluster2028.msf) |  |  |  |  |  |  |  |  |  |  |  |  | |  |  |
| *fixI* | | [43](http://ccg.murdoch.edu.au/organism/rhizobium/RhizobiumSummaries/queryClusters/msf/Cluster43.msf) |  |  |  |  |  |  |  |  |  |  |  |  | |  |  |
| *fixS* | | [19875](http://ccg.murdoch.edu.au/organism/rhizobium/RhizobiumSummaries/queryClusters/seqs/Cluster19875.fa) |  |  |  |  |  |  |  |  |  |  |  |  | |  |  |
| *fixS* | | [19241](http://ccg.murdoch.edu.au/organism/rhizobium/RhizobiumSummaries/queryClusters/seqs/Cluster19241.fa) |  |  |  |  |  |  |  |  |  |  |  |  | |  |  |
| *fixS* | | [17492](http://ccg.murdoch.edu.au/organism/rhizobium/RhizobiumSummaries/queryClusters/seqs/Cluster17492.fa) |  |  |  |  |  |  |  |  |  |  |  |  | |  |  |
| *fixS* | | [12559](http://ccg.murdoch.edu.au/organism/rhizobium/RhizobiumSummaries/queryClusters/seqs/Cluster12559.fa) |  |  |  |  |  |  |  |  |  |  |  |  | |  |  |
| *fixS* | | [11400](http://ccg.murdoch.edu.au/organism/rhizobium/RhizobiumSummaries/queryClusters/seqs/Cluster11400.fa) |  |  |  |  |  |  |  |  |  |  |  |  | |  |  |
| *fixS* | | [13951](http://ccg.murdoch.edu.au/organism/rhizobium/RhizobiumSummaries/queryClusters/seqs/Cluster13951.fa) |  |  |  |  |  |  |  |  |  |  |  |  | |  |  |
| *fixS* | | [17933](http://ccg.murdoch.edu.au/organism/rhizobium/RhizobiumSummaries/queryClusters/seqs/Cluster17933.fa) |  |  |  |  |  |  |  |  |  |  |  |  | |  |  |
| *fixS* | | [17625](http://ccg.murdoch.edu.au/organism/rhizobium/RhizobiumSummaries/queryClusters/seqs/Cluster17625.fa) |  |  |  |  |  |  |  |  |  |  |  |  | |  |  |
| *fixS* | | [16566](http://ccg.murdoch.edu.au/organism/rhizobium/RhizobiumSummaries/queryClusters/seqs/Cluster16566.fa) |  |  |  |  |  |  |  |  |  |  |  |  | |  |  |
| *fixS* | | [16511](http://ccg.murdoch.edu.au/organism/rhizobium/RhizobiumSummaries/queryClusters/seqs/Cluster16511.fa) |  |  |  |  |  |  |  |  |  |  |  |  | |  |  |
| *fixS* | | [20466](http://ccg.murdoch.edu.au/organism/rhizobium/RhizobiumSummaries/queryClusters/seqs/Cluster20466.fa) |  |  |  |  |  |  |  |  |  |  |  |  | |  |  |
| *fixS* | | [21060](http://ccg.murdoch.edu.au/organism/rhizobium/RhizobiumSummaries/queryClusters/seqs/Cluster21060.fa) |  |  |  |  |  |  |  |  |  |  |  |  | |  |  |
| *fixT* | | [9684](http://ccg.murdoch.edu.au/organism/rhizobium/RhizobiumSummaries/queryClusters/msf/Cluster9684.msf) |  |  |  |  |  |  |  |  |  |  |  |  | |  |  |
| *fixU* | | [4485](http://ccg.murdoch.edu.au/organism/rhizobium/RhizobiumSummaries/queryClusters/msf/Cluster4485.msf) |  |  |  |  |  |  |  |  |  |  |  |  | |  |  |
| *fixU* | | [5061](http://ccg.murdoch.edu.au/organism/rhizobium/RhizobiumSummaries/queryClusters/msf/Cluster5061.msf) |  |  |  |  |  |  |  |  |  |  |  |  | |  |  |
| *fixW* | | [20730](http://ccg.murdoch.edu.au/organism/rhizobium/RhizobiumSummaries/queryClusters/seqs/Cluster20730.fa) |  |  |  |  |  |  |  |  |  |  |  |  | |  |  |
|  | *fixABCX* operon - involved in transcription regulation under low O2 i.e in bacteroid form within the nodule. | | | | | | | | | | | | | |  |  |  |
|  | *fixNOPQ* operon - cytochrome cbb3 oxidase. Crucial for oxygen regulation between bacteroid and host plant root cell. | | | | | | | | | | | | | |  |  |  |
|  | *fixQ* = Cytochrome cbb3 subunit IV. Differs signifcantly in different species hence multiple clusters. | | | | | | | | | | | | | |  |  |  |
|  | *fixGHIS* operon - believed to be required for construction of cbb3 oxidase from fix NOPQ components | | | | | | | | | | | | | |  |  |  |

* The gray color indicates absence of the gene.

**Table S18.** Nif orthologs.

| **Coding gene** | **Cluster** | **azc** | **bja** | **bbt** | **bra** | **mlo** | **mes** | **ret** | **rec** | **rlg** | **rlt** | **rle** | **rhi** | **smd** | **sme** |
| --- | --- | --- | --- | --- | --- | --- | --- | --- | --- | --- | --- | --- | --- | --- | --- |
| *nifA* | [30](http://ccg.murdoch.edu.au/organism/rhizobium/RhizobiumSummaries/queryClusters/msf/Cluster30.msf) |  |  |  |  |  |  |  |  |  |  |  |  |  |  |
| *nifB* | [1381](http://ccg.murdoch.edu.au/organism/rhizobium/RhizobiumSummaries/queryClusters/msf/Cluster1381.msf) |  |  |  |  |  |  |  |  |  |  |  |  |  |  |
| *nifD* | [190](http://ccg.murdoch.edu.au/organism/rhizobium/RhizobiumSummaries/queryClusters/msf/Cluster190.msf) |  |  |  |  |  |  |  |  |  |  |  |  |  |  |
| *nifH* | [307](http://ccg.murdoch.edu.au/organism/rhizobium/RhizobiumSummaries/queryClusters/msf/Cluster307.msf) |  |  |  |  |  |  |  |  |  |  |  |  |  |  |
| *nifK* | [213](http://ccg.murdoch.edu.au/organism/rhizobium/RhizobiumSummaries/queryClusters/msf/Cluster213.msf) |  |  |  |  |  |  |  |  |  |  |  |  |  |  |
| *nifE* | [190](http://ccg.murdoch.edu.au/organism/rhizobium/RhizobiumSummaries/queryClusters/msf/Cluster190.msf) |  |  |  |  |  |  |  |  |  |  |  |  |  |  |
| *nifN* | [213](http://ccg.murdoch.edu.au/organism/rhizobium/RhizobiumSummaries/queryClusters/msf/Cluster213.msf) |  |  |  |  |  |  |  |  |  |  |  |  |  |  |
| *nifX* | [2237](http://ccg.murdoch.edu.au/organism/rhizobium/RhizobiumSummaries/queryClusters/msf/Cluster2237.msf) |  |  |  |  |  |  |  |  |  |  |  |  |  |  |
| *nifL** | [168](http://ccg.murdoch.edu.au/organism/rhizobium/RhizobiumSummaries/queryClusters/msf/Cluster168.msf) |  |  |  |  |  |  |  |  |  |  |  |  |  |  |
| *nifQ* | [2971](http://ccg.murdoch.edu.au/organism/rhizobium/RhizobiumSummaries/queryClusters/msf/Cluster2971.msf) |  |  |  |  |  |  |  |  |  |  |  |  |  |  |
| *nifR* | [1362](http://ccg.murdoch.edu.au/organism/rhizobium/RhizobiumSummaries/queryClusters/msf/Cluster1362.msf) |  |  |  |  |  |  |  |  |  |  |  |  |  |  |
| *nifS* | [346](http://ccg.murdoch.edu.au/organism/rhizobium/RhizobiumSummaries/queryClusters/msf/Cluster346.msf) |  |  |  |  |  |  |  |  |  |  |  |  |  |  |
| *nifT*** | [9684](http://ccg.murdoch.edu.au/organism/rhizobium/RhizobiumSummaries/queryClusters/msf/Cluster9684msf) |  |  |  |  |  |  |  |  |  |  |  |  |  |  |
| *nifT*** | [4485](http://ccg.murdoch.edu.au/organism/rhizobium/RhizobiumSummaries/queryClusters/msf/Cluster4485.msf) |  |  |  |  |  |  |  |  |  |  |  |  |  |  |
| *nifT*** | [5061](http://ccg.murdoch.edu.au/organism/rhizobium/RhizobiumSummaries/queryClusters/msf/Cluster5061.msf) |  |  |  |  |  |  |  |  |  |  |  |  |  |  |
| *nifU* | [1606](http://ccg.murdoch.edu.au/organism/rhizobium/RhizobiumSummaries/queryClusters/msf/Cluster1606.msf) |  |  |  |  |  |  |  |  |  |  |  |  |  |  |
| *nifW* | [2972](http://ccg.murdoch.edu.au/organism/rhizobium/RhizobiumSummaries/queryClusters/msf/Cluster2972.msf) |  |  |  |  |  |  |  |  |  |  |  |  |  |  |
| *nifZ* | [2970](http://ccg.murdoch.edu.au/organism/rhizobium/RhizobiumSummaries/queryClusters/msf/Cluster2970.msf) |  |  |  |  |  |  |  |  |  |  |  |  |  |  |

|  | Nitrogenase complex*. nifE* and *nifD* stuctually homologous to *nifN* and *nifK* respectively |  |
| --- | --- | --- |
| * *nifL* - diguanylate cyclase with PAS/PAC sensor only identified as such in rlt. nifL region spans most of the protein. nifL is a negative nif regulator. It is in a cluster shared by all 13 Fix+ genomes entitled “noproteinhit”. All proteins in cluster have various combinations of PAS and CCGF/Diguanylate cyclase domains. | | |
| ** *nifT* also know as *fixU* or *fixT* | | |
| * The gray color indicates absence of the gene. | | |
